# The impact of the world’s first regulatory, multi-setting intervention on sedentary behaviour among children and adolescents (ENERGISE): a natural experiment evaluation

**DOI:** 10.1186/s12966-024-01591-w

**Published:** 2024-05-13

**Authors:** Bai Li, Selene Valerino-Perea, Weiwen Zhou, Yihong Xie, Keith Syrett, Remco Peters, Zouyan He, Yunfeng Zou, Frank de Vocht, Charlie Foster

**Affiliations:** 1 Centre for Exercise, Nutrition and Health Sciences, School for Policy Studies, University of Bristol, Bristol, UK; 2 Public Health Wales, Cardiff, UK; 3 Department of Nutrition and School Health, Guangxi Center for Disease Control and Prevention, Nanning, Guangxi China; 4 School of Public Health, Guangxi Medical University, Nanning, Guangxi China; 5 Centre for Health, Law, and Society, School of Law, University of Bristol, Bristol, UK; 6 Population Health Sciences, Bristol Medical School, University of Bristol, Bristol, UK; 7 NIHR Applied Research Collaboration West (ARC West), Bristol, UK

**Keywords:** Sedentary behaviour, Physical activity, Regulatory intervention, Health policy, Screen time, Natural experiment, Mental health, Well-being, Health promotion, Child health

## Abstract

**Background:**

Regulatory actions are increasingly used to tackle issues such as excessive alcohol or sugar intake, but such actions to reduce sedentary behaviour remain scarce. World Health Organization (WHO) guidelines on sedentary behaviour call for system-wide policies. The Chinese government introduced the world’s first nation-wide multi-setting regulation on multiple types of sedentary behaviour in children and adolescents in July 2021. This regulation restricts when (and for how long) online gaming businesses can provide access to pupils; the amount of homework teachers can assign to pupils according to their year groups; and when tutoring businesses can provide lessons to pupils. We evaluated the effect of this regulation on sedentary behaviour safeguarding pupils.

**Methods:**

With a natural experiment evaluation design, we used representative surveillance data from 9- to 18-year-old pupils before and after the introduction of the regulation, for longitudinal (*n* = 7,054, matched individuals, primary analysis) and repeated cross-sectional (*n* = 99,947, exploratory analysis) analyses. We analysed pre-post differences for self-reported sedentary behaviour outcomes (total sedentary behaviour time, screen viewing time, electronic device use time, homework time, and out-of-campus learning time) using multilevel models, and explored differences by sex, education stage, residency, and baseline weight status.

**Results:**

Longitudinal analyses indicated that pupils had reduced their mean total daily sedentary behaviour time by 13.8% (95% confidence interval [CI]: -15.9 to -11.7%, approximately 46 min) and were 1.20 times as likely to meet international daily screen time recommendations (95% CI: 1.01 to 1.32) one month after the introduction of the regulation compared to the reference group (before its introduction). They were on average 2.79 times as likely to meet the regulatory requirement on homework time (95% CI: 2.47 to 3.14) than the reference group and reduced their daily total screen-viewing time by 6.4% (95% CI: -9.6 to -3.3%, approximately 10 min). The positive effects were more pronounced among high-risk groups (secondary school and urban pupils who generally spend more time in sedentary behaviour) than in low-risk groups (primary school and rural pupils who generally spend less time in sedentary behaviour). The exploratory analyses showed comparable findings.

**Conclusions:**

This regulatory intervention has been effective in reducing total and specific types of sedentary behaviour among Chinese children and adolescents, with the potential to reduce health inequalities. International researchers and policy makers may explore the feasibility and acceptability of implementing regulatory interventions on sedentary behaviour elsewhere.

**Supplementary Information:**

The online version contains supplementary material available at 10.1186/s12966-024-01591-w.

## Background

The growing prevalence of sedentary behaviour in school-aged children and adolescents bears significant social, economic and health burdens in China and globally [1]–[3]. Sedentary behaviour refers to any waking behaviour characterised by an energy expenditure equal or lower than 1.5 metabolic equivalents (METs) while sitting, reclining, or lying [3]. Evidence from systematic reviews, meta-analyses and longitudinal studies have shown that excessive sedentary behaviour, in particular recreational screen-based sedentary behaviour, affect multiple dimensions of children and adolescents’ wellbeing, spanning across mental health [4], cognitive functions/developmental health/academic performance [5], [6], quality of life [7], and physical health [8]. In China, over 60% of school pupils use part of their sleep time to play mobile phones/digital games and watch TV programmes, and 27% use their sleep time to do homework or other learning activities [9]. Screen-based, sedentary entertainment has become the leading cause for going to bed late, which is linked to detrimental consequences for children’s physical and mental health [10]. Notably, academic-related activities such as post-school homework and off campus tutoring also contribute to the increasing amounts of sedentary behaviour. According to the Organisation for Economic Co-operation and Development (OECD) report, China is the leading country in time spent on homework by adolescents (14 h/week on average) [11].

The COVID-19 pandemic exacerbated this global challenge, with children and adolescents reported to have been the most affected group [12]. Schools are a frequently targeted setting for interventions to reduce sedentary behaviour [13]. However, school-based interventions have had limited success when delivered under real-world conditions or at scale [14]. School-based interventions alone have also been unsuccessful in mitigating the trend of increasing sedentary behaviour that is driven by a complex system of interdependent factors across multiple sectors [13]. Even for parents and carers who intend to restrict screen-based sedentary behaviour and for children who wish to reduce screen-based sedentary behaviour, social factors including peer pressure often form barriers to changing behaviour [15]. In multiple public health fields such as tobacco control and healthy eating promotion, there has been a notable shift away from downstream (e.g., health education) towards an upstream intervention approach (e.g., sugar taxation). However, regulatory actions for sedentary behaviour are scarce [16]. World Health Organization (WHO) 2020 guidelines on sedentary behaviour encourage sustainable and scalable approaches for limiting sedentary behaviour and call for more system-wide policies to improve this global challenge [8]. Up-stream interventions can act on sedentary behaviour more holistically and have the potential to maximise reach and health impact [13]. In response to this pressing issue, and to widespread demands from many parents/carers, the Chinese government introduced nationwide regulations in 2021 to restrict (i) the amount of homework that teachers can assign, (ii) when (and for how long) online gaming businesses can provide access to young people, and (iii) when tutoring businesses can provide lessons [17], [18]. Consultations with WHO officials and reviewers of international health policy interventions confirmed that this is currently the only government-led, multi-setting regulatory intervention on multiple types of sedentary behaviour among school-aged children and adolescents. A detailed description of this programme is available in the Additional File 1.

We evaluated the impact of this regulatory intervention on sedentary behaviour in Chinese school-aged children and adolescents. We also investigated whether and how intervention effects differed by sex, education stage, geographical area, and baseline weight status.

## Methods

### Study design

The introduction of the nationwide regulation provided a unique opportunity for a natural experiment evaluation where the pre-regulation comparator group data (Wave 1) was compared to the post-regulation group data (Wave 2). Multiple components of the intervention (see Additional File 1) were introduced in phases from July 2021 with all components being fully in place by September 2021 [17], [18]. This paper follows the STROBE reporting guidance [19], [20].

### Data source, study population and sampling

**Fig. 1:**
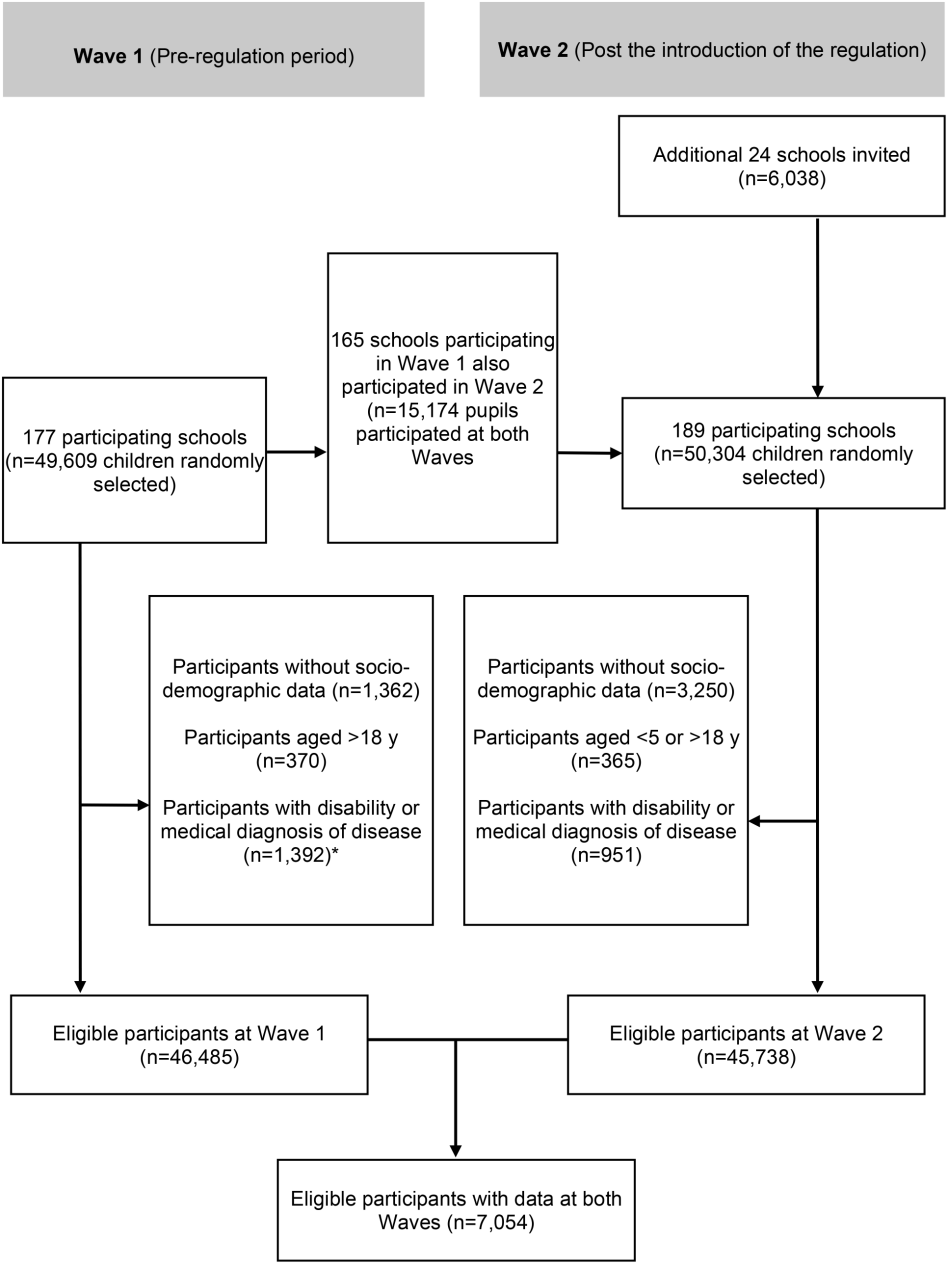
Flow diagram of participants included in the ENERGISE study

We obtained regionally representative data on 99,947 pupils who are resident in the Chinese province of Guangxi as part of Guangxi Centre for Disease Control and Prevention’s (CDC) routine surveillance. The data, available from participants in grade 4 (aged between 9 and 10 years) and higher, were collected using a multi-stage random sampling design (Fig. 1) through school visits by trained health professionals following standardised protocols (see Supplementary Fig. 1, Additional File 1). In Wave 1 (data collected from September to November 2020), pupils were randomly selected from schools in 31 urban/rural counties from 14 cities in Guangxi. At least eight schools, including primary, secondary, high schools, and ‘vocational high schools’, were selected from urban counties. Five schools were selected from rural counties. Approximately 80 students were randomly selected from each grade at the schools selected. The same schools were invited to participate in Wave 2 (data collected from September to November 2021), and new schools were invited to replace Wave 1 schools that no longer participated. Children with available data at both Wave 1 and Wave 2 represented approximately 10% of the sample (*n* = 7,587). Paper-based questionnaires were administrated to students by trained personnel or teachers. The questionnaires were designed and validated by China National Health Commission, and have been utilised in routine surveillance throughout the country.

We used data from the age groups 7–18 years for most analyses. For specific analyses of homework and out-of-campus tutoring, we excluded high school pupils (16–18 years) because the homework and out-of-campus tutoring regulations apply to primary (7–12 years) and middle (13–15 years) school pupils only. Furthermore, participants without socio-demographic data or those who reported medical history of disease, or a physical disability were excluded. This gave us a total sample of 7,054 eligible school-aged children and adolescents with matching data (longitudinal sample).

### Outcomes and subgroups

Guangxi CDC used purposively designed questions for surveillance purposes to assess sedentary behaviour outcomes (Table 1).

**Table 1:** A description of included primary and secondary outcomes

Name outcome variable	Description
**Primary outcomes**	
Total sedentary behaviour time	Sum of ‘total screen-viewing time’ (see secondary outcomes section below), ‘homework time’, and ‘out-of-campus learning time’.
Homework time	Average hours spent per day doing homework, reading, and writing after school in the past week.
Out-of-campus learning time	Average hours spent per day in fee-paying tutoring classes (such as English, math, and writing) in the past week.
Electronic device use time (main estimator of online game time [estimand^*^])	Average hours and minutes spent per day using mobile phones, handheld game consoles, and tablets in the past week.
**Secondary outcomes**	
Total screen-viewing time (alternative estimator of online game time [estimand^*^])	Sum of ‘electronic device use time’ (see primary outcome section above), ‘TV/video game use time’ and ‘computer use time’. ‘TV/video game use time’: Children were asked to report the number of hours per day spent watching TV (including use of game consoles such as the X-BOX) in the past week. ‘Computer-use time’: Children were asked to report the number of hours per day spent using computers in the past week.
Internet use time	Number of hours per day spent being “online” in the past week.
Meeting screen-viewing time recommendations	Engaging in screen-related activities less than 2 h per day as indicated by the American Academy of Paediatrics guidelines^21^.
Meeting regulatory requirement on homework time	Spending less than 60 min per day doing homework for primary-school children and 90 min per day for secondary-school children, as required by the national regulation.

*Estimand is defined as a parameter in the population which is to be estimated in a statistical analysis [22]

The primary outcomes of interest included: (1) total sedentary behaviour time, (2) homework time, (3) out-of-campus learning (private tutoring) time, and (4) electronic device use time (Table 1). We considered electronic device use time, including mobile phones, handheld game consoles, and tablets, the most suitable estimator of online game time (estimand) in the surveillance programme since these are the main devices used for online gaming in China [23]. Secondary outcomes were: (1) total screen-viewing time, (2) internet-use time, (3) likelihood of meeting international screen-viewing time recommendations, and (4) likelihood of meeting the regulation on homework time (Table 1).

We calculated total sedentary behaviour time as the sum of total screen-viewing time (secondary outcome), homework time, and out-of-campus learning time (Table 1). Total screen-viewing time represents the sum of electronic device use time per day, TV/video game use time per day, and computer use time per day (Table 1). Total screen-viewing time was considered as an alternative estimator of online game time (estimand) since TV/videogame console use time and computer time could also capture the small proportion of children who use these devices for online gaming (Table 1). The international screen-viewing time recommendations were based on the American Academy of Paediatrics guidelines [21]. We did not include internet use time (secondary outcome) in total screen-viewing time, and total sedentary behaviour time, because this measure likely overlaps with other variables.

We defined subgroups by demographic characteristics, including the child’s sex (at birth: girls or boys), date of birth, education stage [primary school or secondary school [including middle school, high school, and ‘occupational schools’]), children’s residency (urban versus rural) and children’s baseline weight status (non-overweight versus overweight/obesity). Each sampling site selected for the survey was classified by the surveillance personnel as urban/rural and as lower-, medium-, or higher-economic level based on the area’s gross domestic product (GDP) per capita. The area’s GDP per capita was measured by the Chinese Centre for Disease Control and Prevention (CDC). Trained personnel also measured height, and weight using calibrated stadiometers and scales. Children’s weight/height were measured with light clothing and no shoes. Measurements during both waves were undertaken when students lived a normal life (no lockdowns, school were opened normally). We classified weight status (normal weight vs. overweight/obesity) according to the Chinese national reference charts [24].

### Statistical analyses

We treated sedentary behaviour values that exceeded 24-hours per day as missing. We did not exclude extreme values for body mass index from the analyses^25^. Additional information, justifications, and results of implausible and missing values can be found in the Supplementary Table 1, Additional File 1.

The assumptions for normality and heteroscedasticity were assessed visually by inspecting residuals. We assessed multicollinearity via variance inflation factors. The outcome variables for linear regression outcomes were transformed using square roots to meet assumptions. We reported descriptive demographic characteristics (age, sex, area of residence, socioeconomic status), weight status, and outcome variables using means (or medians for non-normally distributed data) and proportions [26]

We ran multilevel models with random effects nested at the school and child levels to compare the outcomes in Wave 1 against Wave 2. We developed separate models for each sedentary behaviour outcome variable. We treated the introduction of the nationwide regulation as the independent binary variable (0 for Wave 1 and 1 for Wave 2). We ran linear models for continuous outcomes, logistic models for binary outcomes, and ordered logistic models for ordinal outcomes in a complete case analysis estimating population average treatment effects [27]. For the main analysis, in which participants had measurements in both Waves (longitudinal sample), only those with non-missing data at both time points were included.

We estimated marginal effects for each sedentary behaviour outcome. With a self-developed directed acyclic graph (DAG) we identified age (continuous), sex (male/female), area of residence (urban/rural), and socioeconomic status (high/medium/low) as confounders (see Supplementary Figs. 2–4, Additional File 1).

We evaluated subgroup effects defined by child’s sex at birth (boys versus girls), child’s stage of education (primary school versus secondary school [including middle school, high school, and ‘occupational schools’]), children’s residency (rural versus urban), and children’s baseline weight status (non-overweight versus overweight/obesity). We also repeated the covariate-adjusted model with interaction terms (between Wave and sex; Wave and child stage of education; Wave and residency; and Wave and weight status). We adjusted for multiple testing using Bonferroni correction (*p* 0.05 divided by the number of performed tests for an outcome). The resulting cut-off point of *p* < 0.005 was used to determine the presence of any interaction effects.

We also conducted exploratory analyses (including subgroup analyses) by evaluating the same models with a representative, cross-sectional sample of 99,947 pupils. This cross-sectional sample included different schools and children at Wave 1 and Wave 2. We therefore used propensity score (PS) weighting to account for sample imbalances in the socio-demographic characteristics. Propensity scores were calculated by conducting a logistic regression, which calculated the likelihood of each individual to be in Wave 2 (dependent variable). Individual’s age, sex, area of residence and the GDP per area were treated as independent variables. Subsequently, inverse probability of treatment weighting was applied to balance the demographic characteristics in the sample in Wave 1 (unexposed to the regulatory intervention) and Wave 2 (exposed to the regulatory intervention). The sample weight for individuals in Wave 1 were calculated using the Eq. 1/ (1-propensity score). The sample weight for individuals in Wave 2 were calculated using the Eq. 1/propensity score [28].

We only ran linear models for continuous outcomes since it was not possible to run PS-weighted multilevel models with this sample size in Stata. We conducted all statistical analyses in Stata version 16.0.

## Results

### Participant sample

In our primary, longitudinal analyses, we analysed data from 7,054 children and adolescents. The mean age was 12.3 years (SD, 2.4) and 3,477 (49.3%) were girls (Table 2). More detailed information on characteristics of subgroups in the longitudinal sample are presented in the Supplementary Tables 2–5, Additional File 2.

**Table 2:** Characteristics of the longitudinal sample with matched data at both Waves

	Characteristics at baseline (*n* = 7,054)
Socio-demographic characteristics	
Age (years) mean (SD)	12.3 (2.4)
Female n (%)	3,477 (49.3)
Secondary school n (%)	3,969 (56.3)
Urban n (%)	4,402 (62.4)
GDP n (%)	
Low	2,361 (33.5)
Medium	2,277 (32.3)
High	2,416 (34.3)
Normal weight^a^ n (%)	5,686 (80.6)
Primary outcomes	
Total sedentary behaviour time (minutes/day), median (IQR)	330 (240)
Electronic device use time (minutes/day), median (IQR)	60 (125)
Homework time (hours/day), n (%)	
0 h	50 (0.8)
< 1 h	1,366 (20.9)
1–2 h	2,709 (41.4)
2–3 h	1,449 (22.2)
≥ 3 h	966 (14.8)
Out-of-campus learning time (hours/day), n (%)	
0 h	4,291 (63.7)
< 1 h	484 (7.2)
1–2 h	767 (11.4)
2–3 h	527 (7.8)
≥ 3 h	672 (10)
Secondary outcomes	
Total screen viewing time (minutes/day), median (IQR)	150 (192)
Internet use time (minutes/day), median (IQR)	90 (150)
Meeting screen-viewing time recommendation^‡^, n (%)	2,499 (35.6)
Meeting regulatory requirement on homework time^§^, n (%)	2,693 (41.2)

*Abbreviatons* IQR, interquartile range; SD, standard deviation; GDP, gross domestic product

^a^ Excludes participants with overweight or obesity

### Primary outcomes

Children and adolescents reported a reduction in their daily mean total sedentary behaviour time by 13.8% (95% CI: -15.9 to -11.7), or 46 min, on average between Waves 1 and 2. Participants were also less likely to report having increased their time spent on homework (adjusted odd ratio/AOR: 0.39; 95% CI: 0.35–0.43) and in out-of-campus learning (AOR: 0.53; 95% CI: 0.47 to 0.59) in Wave 2 in comparison to Wave 1, respectively (Tables 3 and 4). We did not find any changes in electronic device use time.

**Table 3:** Percentage changes in sedentary behaviours of participants taking part in both waves (main, longitudinal analyses)

	Comparison between Wave 1 and Wave 2				Interaction effect (interaction with Wave)		
	n	Estimate	95% CI	*p-*value^a^	Estimate	95% CI	*p-*value^a^
**Primary outcomes**							
Total sedentary behaviour time^b^							
Model 1^c^	5,959	-10.9	(-12.6, -9.1)	< 0.0001			
Model 2^d^	5,959	-13.8	(-15.9, -11.7)	< 0.0001			
Sex interaction^e^	5,959	-14.2	(-17.0, -11.4)	< 0.0001	0.7	(-2.8, 4.3)	0.68
Education level interaction^f^	5,959	-15.0	(-17.9, -12.2)	< 0.0001	2.2	(-1.3, 5.7)	0.226
Residency interaction^g^	5,959	-10.0	(-13.1, -6.8)	< 0.0001	-6.1	(-9.8, -2.4)	0.0013
Weight status interaction^h^	5,959	-13.7	(-15.9, -11.4)	< 0.0001	-0.7	(-4.2, 2.8)	0.69
Electronic device use time^i^							
Model 1^c^	7,245	4.9	(1.1, 8.7)	0.011			
Model 2^d^	7,245	-3.7	(-8.5, 1.0)	0.13			
Sex interaction^e^	7,245	-4.1	(-10.3, 2.2)	0.20	0.7	(-7.3, 8.6)	0.87
Education stage interaction^f^	7,245	2.9	(-3.5, 9.4)	0.37	-11.9	(-20.1, -3.8)	0.0040
Residency interaction^g^	7,245	1.9	(-5.0, 8.8)	0.59	-9.1	(-17.5, -0.7)	0.033
Weight status interaction^h^	7,245	-4.1	(-9.1, 0.9)	0.11	1.8	(-6.1, 9.6)	0.66
**Secondary outcomes**							
Total screen-viewing time^j^							
Model 1^c^	7,244	-2.5	(-5.1, 0.0)	0.047			
Model 2^d^	7,244	-6.4	(-9.6, -3.3)	0.0001			
Sex interaction^e^	7,244	-8.1	(-12.2, -4.0)	0.0001	3.2	(-1.9, 8.3)	0.22
Education stage interaction^f^	7,244	2.0	(-2.1, 6.2)	0.34	-15.3	(-20.5, -10.0)	< 0.0001
Residency interaction^g^	7,244	-3.1	(-7.6, 1.4)	0.18	-5.4	(-10.8, -0.1)	0.047
Weight status interaction^h^	7,244	-7.3	(-10.6, -4)	< 0.0001	4.3	(-0.8, 9.4)	0.097
Internet use time^k^							
Model 1^c^	4,056	0.8	(-4.3, 6.0)	0.75			
Model 2^d^	4,056	-2.5	(-8.8, 3.8)	0.44			
Sex interaction^e^	4,056	1.2	(-6.9, 9.3)	0.77	-7.7	(-18.5, 3.1)	0.16
Education level interaction^f^	NA	NA	NA	NA	NA	NA	NA
Residency interaction^g^	4,056	7.6	(-2.7, 17.9)	0.15	-14.4	(-26.3, -2.5)	0.018
Weight status interaction^h^	4,056	-2.8	(-9.4, 3.7)	0.40	2.0	(-9.1, 13.1)	0.73

*Abbreviations* CI, confidence interval; NA, non-applicable. The results in this table represent population average treatment effects

^a^Significance assessed at *p* < 0.005 using the Bonferroni correction

^b^Calculated in minutes as the sum of self-reported electronic device use time per day, TV/video game use time per day, computer use time per day, homework time, and out-of-campus learning time

^c^Unadjusted model

^d^Model 1 + age, sex, socioeconomic status, and area of residence

^e^Model 2 + Wave-sex (boys vs. girls) interaction. Reference group are girls/Wave 1

^f^Model 2 + Wave-education stage (primary school vs. secondary school) interaction. Reference group are primary school students/Wave 1

^g^Model 2 + Wave-residency (urban vs. rural) interaction. Reference group are students living in rural areas/Wave 1

^h^Model 2 + Wave-weight status (normal weight vs. overweight/obesity) interaction. Reference group are participants with normal weight/Wave 1

^i^Calculated in minutes from self-reported average time (hours and minutes) per day spent using mobile phones, handheld game consoles, and tablets

^j^Total screen-viewing time calculated as the sum of self-reported electronic device use time per day, TV/video game use time per day, and computer use time per day

^k^Self-reported average time (hours and minutes) per day spent ‘online’; only measured in secondary school students

**Table 4:** Other changes in sedentary behaviours of participants taking part in both waves (main, longitudinal analyses)

	Comparison between Wave 1 and Wave 2				Interaction effect (interaction with Wave)		
	n	Estimate (OR)	95% CI	*p* value^a^	Estimate (OR)	95% CI	*p* value^a^
**Primary outcomes**							
Homework time ^bc^							
Model 1^d^	4,957	0.42	(0.39, 0.46)	< 0.0001			
Model 2^e^	4,957	0.39	(0.35, 0.43)	< 0.0001			
Sex interaction^f^	4,957	0.41	(0.36, 0.46)	< 0.0001	0.90	(0.77, 1.06)	0.20
Education stage interaction^g^	4,957	0.27	(0.24, 0.31)	< 0.0001	2.14	(1.83, 2.50)	< 0.0001
Residency interaction^h^	4,957	0.44	(0.38, 0.50)	< 0.0001	0.82	(0.70, 0.96)	0.013
Weight status interaction^i^	4,957	0.39	(0.35, 0.43)	< 0.0001	1.00	(0.87, 1.16)	0.96
Out-of-campus learning time ^c, j^							
Model 1^d^	5,159	0.52	(0.47, 0.57)	< 0.0001			
Model 2^e^	5,159	0.53	(0.47, 0.59)	< 0.0001			
Sex interaction^f^	5,159	0.53	(0.46, 0.62)	< 0.0001	0.99	(0.83, 1.19)	0.94
Education stage interaction^g^	5,159	0.50	(0.44, 0.57)	< 0.0001	1.18	(0.98, 1.42)	0.078
Residency interaction^h^	5,159	0.58	(0.49, 0.69)	< 0.0001	0.86	(0.71, 1.04)	0.12
Weight status interaction^i^	5,159	0.55	(0.49, 0.63)	< 0.0001	0.82	(0.69, 0.97)	0.024
**Secondary outcomes**							
Meeting screen-viewing time recommendations ^k, l^							
Model 1^d^	7,244	1.07	(0.98, 1.16)	0.11			
Model 2^e^	7,244	1.20	(1.09, 1.32)	< 0.0001			
Sex interaction^f^	7,244	1.25	(1.10, 1.42)	< 0.0001	0.92	(0.78, 1.08)	0.31
Education stage interaction^g^	7,244	0.92	(0.81, 1.04)	0.17	1.73	(1.47, 2.04)	< 0.0001
Residency interaction^h^	7,244	1.06	(0.92, 1.21)	0.44	1.23	(1.04, 1.45)	0.018
Weight status interaction^i^	7,244	1.25	(1.13, 1.38)	< 0.001	0.80	(0.68, 0.94)	0.007
Meeting regulatory requirement on homework time^c, k^							
Model 1^d^	4,957	2.66	(2.41, 2.94)	< 0.0001			
Model 2^e^	4,957	2.79	(2.47, 3.14)	< 0.0001			
Sex interaction^f^	4,957	2.73	(2.34, 3.19)	< 0.0001	1.04	(0.86, 1.25)	0.69
Education stage interaction^g^	4,957	3.55	(3.06, 4.11)	< 0.0001	0.58	(0.48, 0.70)	< 0.0001
Residency interaction^h^	4,957	2.58	(2.19, 3.04)	< 0.0001	1.14	(0.94, 1.38)	0.18
Weight status interaction^i^	4,957	2.79	(2.46, 3.16)	< 0.0001	1.00	(0.84, 1.18)	0.98

*Abbreviations* CI, confidence interval; OR, odds ratio. The results in this table represent population average treatment effects

^a^Significance assessed at *p* < 0.005 using the Bonferroni correction

^b^Homework time calculated from self-reported categories of time spent doing homework

^c^OR for being one category up of time spent doing homework/out-of-campus learning (i.e., spending more time on these activities)

^d^Unadjusted model

^e^Model 1 + age, sex, socioeconomic status (GDP per area), and area of residence

^f^Model 2 + Wave-sex (boys vs. girls) interaction. Reference group are girls/Wave 1

^g^Model 2 + Wave-education stage (primary school vs. secondary school) interaction. Reference group are primary school students/Wave 1

^h^Model 2 + Wave-residency (urban vs. rural) interaction. Reference group are rural areas/Wave 1

^i^Model 2 + Wave-weight status (normal weight vs. overweight/obesity) interaction. Reference group are participants with normal weight/Wave 1

^j^Calculated in self-reported categories of time spent in tutorial classes like English, math, and writing

^k^OR for meeting screen-viewing recommendations and homework time regulatory requirement

^l^Total screen-viewing time calculated as the sum of self-reported electronic device use time per day, TV/video game use time per day, and computer use time per day

### Secondary outcomes

Participants reported reducing their mean daily screen-viewing time by 6.4% (95% CI: -9.6 to -3.3%), or 10 min, on average (Tables 3 and 4). Participants were also 20% as likely to meet international screen time recommendations (AOR: 1.20; 95% CI: 1.09 to 1.32) and were 2.79 times as likely to meet the regulatory requirement on homework time (95% CI: 2.47 to 3.14) compared to the reference group (before the introduction of the regulation).

### Subgroup analyses

Most screen- and study-related sedentary behaviour outcomes differed by education stage (*p* < 0.005) (see Supplementary Tables 6–13, Additional File 2), with the reductions being larger in secondary school pupils than in primary school pupils (Tables 3 and 4, and Table 5). Only secondary school pupils reduced their total screen-viewing time (-8.4%; 95% CI: -12.4 to -4.3) and were also 1.41 times as likely to meet screen-viewing recommendations (AOR: 1.41; 95% CI: 1.23 to 1.61) at Wave 2 compared to Wave 1.

**Table 5:** Changes in sedentary behaviours of participants taking part in both waves by subgroups (main, longitudinal analyses)

	Primary outcomes^a^ (Estimate, 95%CI)				Secondary outcomes^a^ (Estimate, 95%CI)			
	Total sedentary behaviour time^b^	Electronic device use time^c^	Homework time^d, e^¶	Out-of-campus learning time^f^	Total screen viewing time^g^	Internet use time^h^	Meeting screen-viewing time recommendations^i^	Meeting regulatory homework time requirement^i^
**Child sex**								
Boys	-14.1 (-16.9, -11.2)	-5.3 (-12.0, 1.4)	0.37 (0.32, 0.42)	0.53 (0.45, 0.62)	-5.5 (-9.8, -1.2)	-7.5 (-16.7, 1.7)	1.11 (0.98, 1.27)	2.73 (2.31, 3.22)
Girls	-14.4 (-17.4, -11.5)	-5.1 (-11.8, 1.6)	0.38 (0.33, 0.43)	0.54 (0.46, 0.64)	-8.5 (-12.8, -4.2)	1.7 (-6.8, 10.2)	1.32 (1.16, 1.51)	2.63 (2.22, 3.12)
**Child education stage**								
Primary school	-19.1 (-22.7, -15.5)	0.8 (-8.6, 10.3)	0.30 (0.26, 0.34)	0.47 (0.40, 0.55)	-2.3 (-7.6, 2.9)	NA	1.02 (0.88, 1.18)	3.61 (3.09, 4.22)
Secondary school	-9.5 (-12.2, -6.8)	-3·6 (-9.3, 2.1)	0.58 (0.50, 0.67)	0.59 (0.49, 0.71)	-8.4 (-12.4, -4.3)	NA	1.41 (1.23, 1.61)	2.11 (1.74, 2.56)
**Child residence**								
Urban	-15.3 (-17.8, -12.7)	-3.4 (-8.9, 2.1)	0.34 (0.30, 0.39)	0.47 (0.41, 0.55)	-6.6 (-10.5, -2.8)	-6.5 (-13.7, 0.7)	1.29 (1.14, 1.46)	3.04 (2.60, 3.56)
Rural	-11.2 (-15.0, -7.4)	-3.5 (-12.7, 5.7)	0.45 (0.39, 0.52)	0.65 (0.54, 0.79)	-6.2 (-11.5, -0.8)	7.0 (-5.4, 19.3)	1.07 (0.92, 1.24)	2.46 (2.05, 2.96)
**Child baseline weight status**								
Normal weight	-13.2 (-15.5, -10.8)	-3.7 (-9.0, 1.6)	0.39 (0.35, 0.44)	0.58 (0.51, 0.66)	-6.7 (-10.2, -3.3)	-4.0 (-10.9, 2.8)	1.24 (1.12, 1.38)	2.70 (2.37, 3.08)
Overweight/obesity	-18.1 (-22.5, -13.8)	-9.4 (-20.1, 1.3)	0.31 (0.25, 0.39)	0.41 (0.32, 0.53)	-7.4 (-14.2, -0.7)	5.1 (-9.6, 19.7)	1.08 (0.89, 1.32)	2.63 (2.03, 3.40)

*Abbreviations* CI, confidence interval; NA, non-applicable; OR, odds ratio. The results in this table represent population average treatment effects

^a^Model 1 including age, sex, GDP per area, and area of residence

^b^Calculated in minutes as the sum of self-reported electronic device use time per day, TV/video game use time per day, computer use time per day, homework time, and out-of-campus learning time. Presented in percentage changes

^c^Calculated in minutes from self-reported average time (hours and minutes) per day spent using mobile phones, handheld game consoles, and tablets. Presented in percentage changes

^d^Homework time calculated from self-reported categories of time spent doing homework

^e^OR for being one category up of time spent doing homework/out-of-campus learning (i.e., spending more time on these activities)

^f^Calculated in self-reported categories of time spent in tutorial classes like English, math, and writing

^g^Screen time calculated as the sum of self-reported electronic device use time per day, TV/video game use time per day, and computer use time per day. Presented in percentage changes

^h^Calculated in minutes from self-reported average time (hours and minutes) per day spent ‘online’; only measured in secondary school children. Presented in percentage changes

^i^OR for meeting screen-viewing recommendations and homework time regulatory requirement

Conversely, at Wave 2, primary school pupils reported a lower likelihood of spending more time doing homework (AOR: 0.30; 95%: 0.26 to 0.34) than secondary school pupils (AOR: 0.58; 95% CI: 0.50 to 0.67) compared to their counterparts at Wave 1. At Wave 2, primary school pupils also had a higher likelihood of reporting meeting homework time recommendations (AOR: 3.61; 95% CI: 3.09 to 4.22) than secondary school pupils (middle- and high school) (AOR: 2.11; 95% CI: 1.74 to 2.56) compared to their counterparts at Wave 1 (Table 5). There was also a residence interaction effect (*p* < 0.001) in total sedentary behaviour time, with participants in urban areas reporting larger reductions (-15.3%; 95% CI: -17.8 to -12.7) than those in rural areas (-11.2%; 95% CI: -15.0 to -7.4). There was no evidence of modifying effects by children’s sex or baseline weight status (Tables 4 and 5).

Findings from the exploratory repeated cross-sectional analyses were similar to the findings of the main longitudinal analyses including total sedentary behaviour time, electronic device use time, total screen-viewing time and internet use time (see Supplementary Tables 14–23, Additional File 2).

## Discussion

### Principal findings

Our study evaluated the impact of the world’s first regulatory, multi-setting intervention on multiple types of sedentary behaviour among school-aged children and adolescents in China. We found that children and adolescents reduced their total sedentary behaviour time, screen-viewing time, homework time and out-of-campus learning time following its implementation. The positive intervention effects on total screen-viewing time (-8.4 vs. -2.3%), and the likelihood of meeting recommendations on screen-viewing time (1.41 vs. 1.02 AOR) were more pronounced in secondary school pupils compared with primary school pupils. Intervention effects on total sedentary behaviour time (-15.3 vs. -11.2%) were more pronounced among pupils living in the urban area (compared to pupils living in the rural area). These subgroup differences imply that the regulatory intervention benefit more the groups known to have a higher rate of sedentary behaviour [29].

Interestingly, the observed reduction in electronic device use itself did not reach statistical significance following implementation of regulation. This could be viewed as a positive outcome if this is correctly inferred and not the result of reporting bias or measurement error. International data indicated that average sedentary and total screen time have increased among children due to the COVID-19 pandemic [12]. However, such interesting finding might be explained by the absence of lockdowns in Guangxi during both surveillance waves when most school-aged students outside China were affected by pandemic mitigation measures such as online learning.

### Strengths and weaknesses

Our study has several notable strengths. This is the first study to evaluate the impact of multi-setting nationwide regulations on multiple types of sedentary behaviour in a large and regionally representative sample of children and adolescents. Still, to gain a more comprehensive view of the regulatory intervention on sedentary behaviour across China, similar evaluation research should be conducted in other regions of China. Furthermore, access to a rich longitudinal dataset allowed for more robust claims of causality. The available data also allowed us to measure the effect of the intervention on multiple sedentary behaviours including recreational screen-time and academic-related behaviours. Lastly, the large data set allowed us to explore whether the effect of the regulatory intervention varied across important subgroups, suggesting areas for further research and development.

Some limitations need to be taken into consideration when interpreting our findings. First, a common limitation in non-controlled/non-randomised intervention studies is residual confounding. We aimed to limit this by adjusting our analysis for confounders known to impact the variables of interest, but it is impossible to know whether important confounding may still have been present. With maturation bias, it is possible that secular trends are the cause for any observed effects. However, this seems unlikely in our study as older children may spend more time doing homework [23] and engage more in screen-viewing activities [30]. In this study, we observed reductions in these outcomes. The use of self-reported outcomes (social desirability bias) was a limitation and might have led to the intervention effects being over-estimated [13]. However, since our data were collected as part of a routine surveillance programme, pupils were unaware of the evaluation. This might mitigate reporting bias. In addition, the data were collected in Guangxi which might not representative of the whole population in China. Another limitation is using electronic device use time as a proxy measure of online gaming time. It is possible that electronic devices can be used for other purposes. However, mobile phones, handheld game consoles and tablets are the main devices used for online gaming. In this study, electronic device use time provided a practical means of assessing the broad effects of regulatory measures on screen time behaviours, including online gaming, in a large (province level) surveillance programme. In the future, instruments specifically designed to capture online gaming behaviour should be used in surveillance and research work.

### Comparisons with other studies

Neither China nor other countries globally have previously implemented and evaluated multi-setting regulatory interventions on multiple types of sedentary behaviour, which makes comparative discussions challenging. In general, results of health behaviour research over the past decades have shown that interventions that address structural and environmental determinants of multiple behaviours to be more effective in comparison with individual-focussed interventions [31]. Furthermore, the continuous and universal elements of regulatory interventions may be particularly important explanations for the observed reductions in sedentary behaviour. Standalone school and other institution-led interventions may struggle with financial and logistic costs which threaten long-term implementation [13]. In contrast, the universality element of regulatory intervention can reduce or remove peer pressures and potential stigmatisation among children and teachers that are often associated with more selective/targeted interventions [24]. Our findings support WHO guidelines for physical activity and sedentary behaviour that encourage sustainable and scalable approaches for limiting sedentary behaviour and call for more system-wide policies to improve this global challenge[8].

### Implications for future policy and research

Our study has important implications for future research and practice both nationally and internationally. Within China, future research should focus on optimising the implementation of the regulatory intervention through implementation research and assess long-term effects of the regulation on both behavioral and health outcomes. Internationally, our findings also provide a promising policy avenue for other countries and communities outside of China to explore the opportunities and barriers to implement such programmes on sedentary behaviour. This exploratory process could start with assessing how key stakeholders (including school-aged children, parents/carers, schoolteachers, health professionals, and policy makers) within different country contexts perceive regulatory actions as an intervention approach for improving health and wellbeing in young people, and how they can be tailored to fit their own contexts. Within public health domains, including healthy eating promotion, tobacco and alcohol control, regulatory intervention approaches (e.g., smoking bans and sugar taxation) have been adopted. However, regulatory actions for sedentary behaviour are scarce [19]. Within the education sector, some countries recently banned mobile phone use in schools for academic purpose [25]. While this implies potential feasibility and desirability of such interventions internationally, there is little research on the demand for, and acceptability of, multi-faceted sedentary behaviour regulatory interventions for the purpose of improving health and wellbeing. It will be particularly important to identify and understand any differences in perceptions and feasibility both within (e.g., public versus policy makers) and across countries of differing socio-cultural-political environments.

## Conclusions

This natural experiment evaluation indicates that a multi-setting, regulatory intervention on sedentary behaviour has been effective in reducing total sedentary behaviour, and multiple types of sedentary behaviour among Chinese school-aged children and adolescents. Contextually appropriate, regulatory interventions on sedentary behaviour could be explored and considered by researchers and policy makers in other countries.

## Electronic supplementary material

Below is the link to the electronic supplementary material.

Supplementary Material 1

Supplementary Material 2

Supplementary Material 3

## Data Availability

Access to anonymised data used in this study can be requested through the corresponding author BL, subject to approval by the Guangxi CDC. WZ and SVP have full access to all the data in the study and takes responsibility for the integrity of the data and the accuracy of the data analysis.

## Abbreviations

CDC: Centre for disease control and prevention
DAG: Directed acyclic graph
GDP: Gross domestic product
METs: Metabolic equivalents
OECD: Organisation for Economic Co-operation and Development
