# Correction: The impact of the world’s first regulatory, multi-setting intervention on sedentary behaviour among children and adolescents (ENERGISE): a natural experiment evaluation

**DOI:** 10.1186/s12966-024-01695-3

**Published:** 2024-12-12

**Authors:** Bai Li, Selene Valerino-Perea, Weiwen Zhou, Yihong Xie, Keith Syrett, Remco Peters, Zouyan He, Yunfeng Zou, Frank de Vocht, Charlie Foster

**Affiliations:** 1 Centre for Exercise, Nutrition and Health Sciences, School for Policy Studies, University of Bristol, Bristol, UK; 2 Public Health Wales, Cardiff, UK; 3 Department of Nutrition and School Health, Guangxi Center for Disease Control and Prevention, Nanning, Guangxi China; 4 School of Public Health, Guangxi Medical University, Nanning, Guangxi China; 5 Centre for Health, Law, and Society, School of Law, University of Bristol, Bristol, UK; 6 Population Health Sciences, Bristol Medical School, University of Bristol, Bristol, UK; 7 NIHR Applied Research Collaboration West (ARC West), Bristol, UK

**Correction to: Li et al. International Journal of Behavioral Nutrition and Physical Activity (2024) 21:53**

10.1186/s12966-024-01591-w

Following the publication of the Original Article, the authors reported that the Funding statement was incomplete as it had yet to mention the relevant grant number [204813/Z/16/Z].

The complete funding statement is as follows:

This work was funded by the Wellcome Trust **[204813/Z/16/Z]** through the Global Public Health Research Strand, Elizabeth Blackwell Institute for Health Research. The funder of our study had no role in the design and conduct of the study; collection, management, analysis, and interpretation of the data; preparation, review, or approval of the manuscript; and decision to submit the manuscript for Publication.

The original article has been corrected.

