# Supplementary material for: The impact of the world’s first regulatory, multi-setting intervention on sedentary behaviour among children and adolescents (ENERGISE): a natural experiment evaluation
Source: Int J Behav Nutr Phys Act. 2024 May 13;21:53. doi: 10.1186/s12966-024-01591-w (PMC11089804; doi:10.1186/s12966-024-01591-w)
Supplement: Supplementary file 1 — Supplementary Material 1 [file 12966_2024_1591_MOESM1_ESM.docx]

Additional File 1

**The impact of the world’s first regulatory**, **multi-setting intervention on sedentary behaviour among children and adolescents (ENERGISE): a natural experiment evaluation**

**Intervention description *(page 2)***

**Methods *(page 2)***

Supplementary Table 1 Percentage of participants with incomplete or implausible data *(page 3)*

Supplementary Figure 1 Overview of the sampling strategy for the surveillance surveys by the Guangxi Centre for Disease Control and Prevention *(page 2)*

Supplementary Figure 2 Directed acyclic graph (DAG) with screen-viewing time as outcome *(page 4)*

Supplementary Figure 3 Directed acyclic graph (DAG) with homework time as outcome *(page 5)*

Supplementary Figure 4 Directed acyclic graph (DAG) with out-of-campus learning time as outcome *(page 6)*

# Intervention description

In 2021, multi-setting nation-wide regulatory actions on sedentary behaviour were introduced by the Chinese government that include the following key components: 1) no written homework to be assigned to students in Grade 1 (6 to 7 years of age) and Grade 2 (7 to 8 years of age); and for students in Grades 3-6 (8 to12 years of age) and Grades 7-9 (12 to 15 years of age), assigned written homework must not exceed 60 and 90 minutes per day, respectively, 2) all existing academic tutoring companies are automatically registered as ‘non-profit making’ businesses and are not allowed to provide paid services on weekends, national holidays, and school vacations; and 3) online game providers must not provide services to children and adolescents below the age of 18 after 22:00 hours and they can only provide services for one hour from 20:00 pm to 21:00 pm on weekend days and during public holidays. This regulation is enforced as under-18s must register and log in to online games using Chinese citizenship card numbers, and online game service providers not adhering to these regulations face financial penalties^1, 2^.

# Methods

**Supplementary Figure 1** Overview of the sampling strategy for the surveillance surveys by the Guangxi Centre for Disease Control and Prevention.

14 cities

Guangxi province

Urban counties at each city

8 schools selected at random at each county:

- 2 primary schools (grades 4-6 only)
- 2 secondary schools
- 2 high schools
- 2 vocational high schools

80 children selected at random from each grade at each school.

Rural counties at each city

5 schools selected at random at each county:

- 2 primary schools (grades 4-6 only)
- 2 secondary schools
- 1 high school

80 children selected at random from each grade at each school.

31 urban/rural counties

## Incomplete and implausible data

**Supplementary Table 1** Percentage of participants with incomplete or implausible data

|  | **Children with matched data at both Waves** | | **Wave 1** | | **Wave 2** | |
| --- | --- | --- | --- | --- | --- | --- |
|  | Incomplete | Implausible* | Incomplete | Implausible* | Incomplete | Implausible* |
| Age | 0.0 | 0.0 | 0.0 | 0.0 | 0.0 | 0.0 |
| Sex | 0.0 | 0.0 | 0.0 | 0.0 | 0.0 | 0.0 |
| Residence | 0.0 | 0.0 | 0.0 | 0.0 | 0.0 | 0.0 |
| Socioeconomic status | 0.0 | 0.0 | 0.0 | 0.0 | 0.0 | 0.0 |
| Grade | 0.0 | 0.0 | 0.0 | 0.0 | 0.0 | 0.0 |
| Weight status | 0.0 | 0.0 | <0.1 | 0.0 | 0.2 | 0.0 |
| Electronic device use | 0.4 | <0.1 | 0.6 | <0.1 | 0.5 | 0.1 |
| Homework time | 6.9 | 0.0 | 7.4 | 0.0 | 7.0 | 0.0 |
| Out-of-campus learning time | 4.1 | 0.0 | 4.3 | 0.0 | 4.4 | 0.0 |
| Sedentary behaviour | 10.4 | <0.1 | 10.9 | <0.1 | 10.5 | 0.1 |
| Screen-viewing time | 0.4 | <0.1 | 0.6 | <0.1 | 0.5 | 0.1 |
| Parental control of screen-viewing time | 0.2 | 0.0 | 0.3 | 0.0 | 0.2 | 0.0 |
| Internet use time | 42.9 | <0.1 | 34.4 | <0.1 | 34.7 | <0.1 |

*Refers to daily self-reported electronic device use, sedentary behaviour, screen-viewing time, and internet use exceeding 24 hours per day. These implausible values were set as missing data.

^†^Only secondary school children were asked to report this variable.

## **Directed acyclic graphs created to identify covariates**


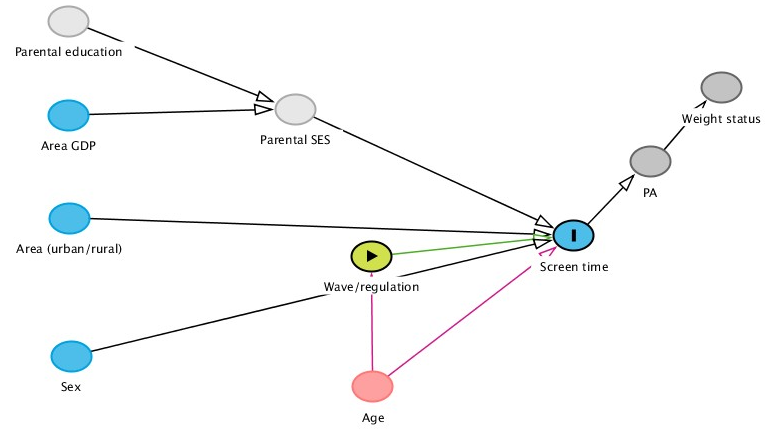


## Supplementary Figure 2 Directed acyclic graph (DAG) with screen-viewing time as outcome

Directed acyclic graph (DAG) drawn from the literature^4-13^ and expert knowledge. Exposure (Wave before or after the introduction of the nationwide regulations on video game time) is presented in green. The outcome, screen-viewing time (SVT), is presented in a blue circle with a line (right-hand side). These regulations are expected to reduce child’s screen-viewing time (mediator). Parental education (unmeasured) and the area’s gross domestic product per capita could determine the parental socioeconomic status (SES; unmeasured), which in turn could determine parental academic expectations (unmeasured) and, therefore, parental control of screen time to increase homework time. Parental SES has also been associated with reduced TV viewing, but higher time spent on video games. Area: living in urban areas has been associated with less TV-viewing time but more ‘computer use time’. Sex: boys spend more time playing video games and in total SVT. Age: older children report higher SVT, although being in transitioning education stages (final years of primary, secondary, and high school) could be a moderator of this association; age is also related to the exposure (Wave) as it differs at Wave 1 vs Wave 2 and therefore considered a confounder.


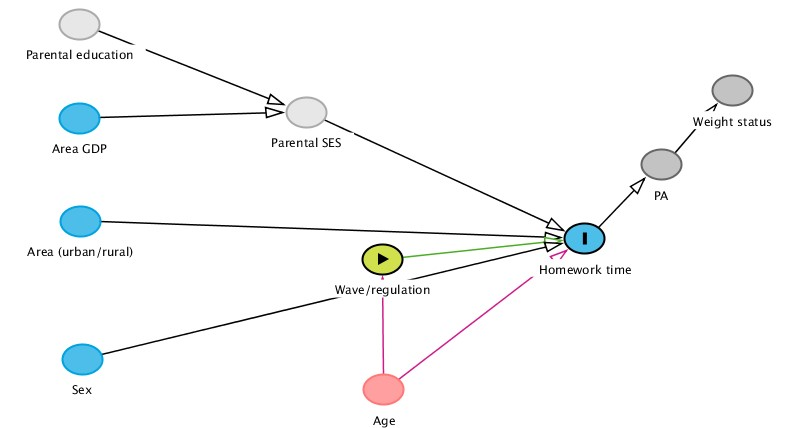


## Supplementary Figure 3 Directed acyclic graph (DAG) with homework time as outcome

Directed acyclic graph (DAG) drawn based on published literature^4-13^, expert knowledge and availability of variables. Exposure [Wave before or after the introduction of the nationwide regulations on homework time (HT)] is presented in green. The outcome, HT, is presented in a blue circle with a line (right-hand side). The introduction of regulations is expected to reduce HT, which could increase physical activity (PA) and, therefore, weight status (explaining negative associations between PA and HT and positive associations between obesity and HT. Parental education (unmeasured) and the area’s gross domestic product per capita could determine the parental socioeconomic status (SES) (unmeasured), both of which have been positively associated with obesity, potentially through higher academic expectations leading to more HT and, therefore, less PA. Area: children living in urban areas spend more time doing homework. Sex: girls spend more time doing homework, although the education stage is a moderator in this association. Age: older children spend more time doing homework, although being in transitioning education stages (final years of primary, secondary, and high school) could be a moderator of this association; age is also related to the exposure (Wave) as it differs at Wave 1 vs Wave 2 and therefore considered a confounder.


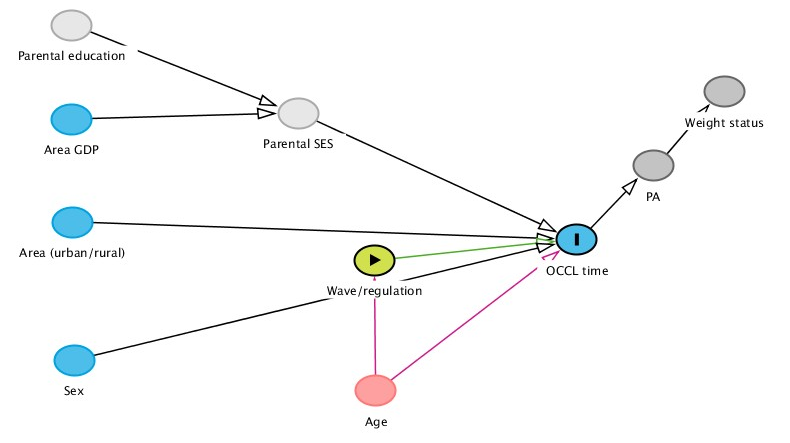


## Supplementary Figure 4 Directed acyclic graph (DAG) with out-of-campus learning time as outcome

Directed acyclic graph (DAG) drawn based on published literature^4-13^, expert knowledge and availability of variables. Exposure [Wave before or after introducing the nationwide regulations on out-of-campus learning time (OOCL time)] is presented in green. The outcome, OOCL time, is presented in a blue circle filled with a line (right-hand side). The introduction of regulations is expected to reduce OOCL time. Parental education (unmeasured) and the area’s gross domestic product per capita could determine the parental socioeconomic status (SES) (unmeasured), both of which could increase academic expectations leading to more OOCL time. Area: children living in urban areas spend more time in OOCL time. Sex: engagement in OOCL time could differ by sex. Age: older children spend more time in OOCL time, although being in transitioning education stages (final years of primary, secondary, and high school) could be a moderator of this association; age is also related to the exposure (Wave) as it differs at Wave 1 vs Wave 2 and therefore considered a confounder.

# Reference list

1. China Internet Network Information Center: **2020年全国未成年人互联网使用情况研究报告 [2020 National Research Report on Internet Use by Minors]**. In*.* Beijing; 2020.

2. **国家新闻出版署关于进一步严格管理 切实防止未成年人沉迷网络游戏的通知 [Notice of the State Press and Publication Administration on Further Strict Management to Effectively Prevent Minors from Being Addicted to Online Games]** [<https://www.gov.cn/zhengce/zhengceku/2021-09/01/content_5634661.htm>]

3. Chesnaye NC, Stel VS, Tripepi G, Dekker FW, Fu EL, Zoccali C, Jager KJ: **An introduction to inverse probability of treatment weighting in observational research**. *Clinical Kidney Journal* 2021, **15**(1):14-20.

4. Huang WY, Wong SH, Salmon J: **Correlates of physical activity and screen-based behaviors in Chinese children**. *Journal of Science and Medicine in Sport* 2013, **16**(6):509-514.

5. Dearth-Wesley T, Howard AG, Wang H, Zhang B, Popkin BM: **Trends in domain-specific physical activity and sedentary behaviors among Chinese school children, 2004–2011**. *International Journal of Behavioral Nutrition and Physical Activity* 2017, **14**(1):141.

6. Li M, Dibley MJ, Sibbritt DW, Zhou X, Yan H: **Physical Activity and Sedentary Behavior in Adolescents in Xi’an City, China**. *Journal of Adolescent Health* 2007, **41**(1):99-101.

7. Zhu X, Haegele JA, Tang Y, Wu X: **Prevalence and Demographic Correlates of Overweight, Physical Activity, and Screen Time Among School-Aged Children in Urban China: The Shanghai Study**. *Asia Pacific Journal of Public Health* 2018, **30**(2):118-127.

8. Zhu X, Haegele JA, Tang Y, Wu X: **Physical Activity and Sedentary Behaviors of Urban Chinese Children: Grade Level Prevalence and Academic Burden Associations**. *BioMed Research International* 2017, **2017**:7540147.

9. Carson V, Hunter S, Kuzik N, Gray CE, Poitras VJ, Chaput J-P, Saunders TJ, Katzmarzyk PT, Okely AD, Connor Gorber S *et al*: **Systematic review of sedentary behaviour and health indicators in school-aged children and youth: an update**. *Applied Physiology Nutrition and Metabolism* 2016, **41**(6 (Suppl. 3)):S240-S265.

10. Zhang T, Cai L, Ma L, Jing J, Chen Y, Ma J: **The prevalence of obesity and influence of early life and behavioral factors on obesity in Chinese children in Guangzhou**. *BMC Public Health* 2016, **16**(1):954.

11. Ren H, Zhou Z, Liu W, Wang X, Yin Z: **Excessive homework, inadequate sleep, physical inactivity and screen viewing time are major contributors to high paediatric obesity**. *Acta Paediatrica* 2017, **106**(1):120-127.

12. Tian X, Wang H: **Growth and Weight Status in Chinese Children and Their Association with Family Environments**. *Children* 2021, **8**(5):397.

13. Li M, Xue H, Wang W, Wang Y: **Parental Expectations and Child Screen and Academic Sedentary Behaviors in China**. *American Journal of Preventive Medicine* 2017, **52**(5):680-689.
