# Supplementary material for: The impact of the world’s first regulatory, multi-setting intervention on sedentary behaviour among children and adolescents (ENERGISE): a natural experiment evaluation
Source: Int J Behav Nutr Phys Act. 2024 May 13;21:53. doi: 10.1186/s12966-024-01591-w (PMC11089804; doi:10.1186/s12966-024-01591-w)
Supplement: Supplementary file 2 — Supplementary Material 2 [file 12966_2024_1591_MOESM2_ESM.docx]

Additional File 2

**The impact of the world’s first regulatory**, **multi-setting intervention on sedentary behaviour among children and adolescents (ENERGISE): a natural experiment evaluation**

**Results of the main analysis based on longitudinal sample (children with matching data at Wave 1 and 2) (page *3)***

**Supplementary Table 2** Sociodemographic and health-related characteristics of the sample with matched data at both Waves, boys in comparison to girls (main analysis) *(page 3)*

**Supplementary Table 3**. Sociodemographic and health-related characteristics of the sample with matched data at both Waves, primary school in comparison to secondary school students (main analysis) *(page 4)*

**Supplementary Table 4**. Sociodemographic and health-related characteristics of the sample with matched data at both Waves, urban in comparison to rural residents (main analysis) *(page 5)*

**Supplementary Table 5**. Sociodemographic and health-related characteristics of the sample with matched data at both Waves, participants with normal weight in comparison to overweigh/obesity at baseline (main analysis) *(page 6)*

**Supplementary Table 6**. Percentage changes in sedentary behaviours of participants taking part in both waves after the introduction of nationwide regulations, boys in comparison to girls *(page 7)*

**Supplementary Table 7**. Odds ratio for being one category up in homework and out-of-campus learning time, and for meeting recommendations, in participants taking part in both waves after the introduction of nationwide regulations, boys in comparison to girls (main analysis) *(page 8)*

**Supplementary Table 8**. Percentage changes in sedentary behaviours of participants taking part in both waves after the introduction of nationwide regulations, primary in comparison to secondary school students (main analysis) *(page 9)*

**Supplementary Table 9**. Odds ratio for being one category up in homework and out-of-campus learning time, and for meeting recommendations taking part in both waves after the introduction of nationwide regulations, primary in comparison to secondary school students (main analysis) *(page 10)*

**Supplementary Table 10**. Percentage changes in sedentary behaviours of participants taking part in both waves after the introduction of nationwide regulations, urban in comparison to rural residents (main analysis) *(page 11)*

**Supplementary Table 11**. Odds ratio for being one category up in homework and out-of-campus learning time, and for meeting recommendations screen-viewing time in participants taking part in both waves after the introduction of nationwide regulations, urban in comparison to rural residents (main analysis) *(page 12)*

**Supplementary Table 12**. Percentage changes in sedentary behaviours of participants taking part in both waves after the introduction of nationwide regulations, participant with normal weight in comparison to participants who have overweight/obesity (main analysis) *(page 13)*

**Supplementary Table 13**. Odds ratio for being one category up in homework and out-of-campus learning time, and for meeting recommendations in participants taking part in both waves after the introduction of nationwide regulations, participant with normal weight in comparison to participants who have overweight/obesity (main analysis) *(page 14)*

**Results of exploratory analysis based on full sample (repeated cross-sectional analyses using representative samples) *(page 15)***

**Supplementary Table 14**. Sociodemographic and health-related characteristics of the complete sample by periods of the introduction of nationwide regulatory interventions (exploratory repeated cross-sectional analyses) *(page 15)*

**Supplementary Table 15**. Sociodemographic and health-related characteristics of the complete sample by periods of the introduction of nationwide regulatory interventions, boys in comparison to girls (exploratory repeated cross-sectional analyses) *(page 16)*

**Supplementary Table 16**. Sociodemographic and health-related characteristics of the complete sample by periods of the introduction of nationwide regulatory interventions, primary school in comparison to secondary school students (exploratory repeated cross-sectional analyses) *(page 17)*

**Supplementary Table 17**. Sociodemographic and health-related characteristics of the complete sample by periods of the introduction of nationwide regulatory interventions, urban in comparison to rural residents (exploratory repeated cross-sectional analyses) *(page18)*

**Supplementary Table 18**. Sociodemographic and health-related characteristics of the complete sample by periods of the introduction of nationwide regulatory interventions, participants with normal weight in comparison to overweigh/obesity (exploratory repeated cross-sectional analyses) *(page 19)*

**Supplementary Table 19**. Percentage changes in sedentary behaviours of participants taking part in both waves after the introduction of nationwide regulations (exploratory repeated cross-sectional analyses) *(page 20)*

**Supplementary Table 20**. Percentage changes in sedentary behaviours of participants after the introduction of nationwide regulations, boys in comparison to girls (exploratory repeated cross-sectional analyses) *(page 22)*

**Supplementary Table 21**. Percentage changes in sedentary behaviours of participants after the introduction of nationwide regulations, primary in comparison to secondary school students (exploratory repeated cross-sectional analyses) *(page 23)*

**Supplementary Table 22**. Percentage changes in sedentary behaviours of participants after the introduction of nationwide regulations, urban in comparison to rural residents (exploratory repeated cross-sectional analyses) *(page 24)*

**Supplementary Table 23**. Percentage changes in sedentary behaviours of participants after the introduction of nationwide regulations, participant with normal weight in comparison to overweight/obesity (exploratory repeated cross-sectional analyses) *(page 25)*

# Results of the main analysis based on longitudinal sample (children with matching data at Wave 1 and 2)

## Supplementary Table 2. Sociodemographic and health-related characteristics of the sample with matched data at both Waves, boys in comparison to girls (main analysis)

|  | Boys (n=3,577) | Girls (n=3,477) |
| --- | --- | --- |
| Socio-demographic characteristics |  |  |
| Age (years), mean (SD) | 12.2 (2.3) | 12.4 (2.4) |
| Secondary school, n (%) | 1,927 (53.9) | 2,042 (58.7) |
| Urban residence, n (%) | 2,288 (64) | 2,114 (60.8) |
| GDP, n (%) |  |  |
| Low | 1,198 (33.5) | 1,163 (33.4) |
| Medium | 1,101 (30.8) | 1,176 (33.8) |
| High | 1,278 (35.7) | 1,138 (32.7) |
| Normal weight^a^, n (%) | 2,693 (75.3) | 2,993 (86.1) |
| Primary outcomes |  |  |
| Total sedentary behaviour time (minutes/day), median (IQR) | 330 (240) | 320 (240) |
| Electronic device use time (minutes/day), median (IQR) | 60 (123) | 60 (130) |
| Homework time (hours/day), n (%) |  |  |
| 0 hours | 24 (0.7) | 26 (0.8) |
| <1 hour | 740 (22.1) | 626 (19.6) |
| 1-2 hours | 1,400 (41.8) | 1,309 (41) |
| 2-3 hours | 719 (21.5) | 730 (22.9) |
| ≥3 hours | 466 (13.9) | 500 (15.7) |
| Out-of-campus learning time (hours/day), n (%) |  |  |
| 0 hours | 2,118 (62.5) | 2173 (64.8) |
| <1 hour | 268 (7.9) | 216 (6.4) |
| 1-2 hours | 393 (11.6) | 374 (11.2) |
| 2-3 hours | 279 (8.2) | 248 (7.4) |
| ≥3 hours | 331 (9.8) | 341 (10.2) |
| Secondary outcomes |  |  |
| Screen-viewing time (minutes/day), median (IQR) | 160 (210) | 150 (200) |
| Meeting screen-viewing time recommendation, n (%) | 1,228 (34.5) | 1,271 (36.7) |
| Meeting regulatory requirement on homework time, n (%) | 1,408 (42) | 1,285 (40.3) |
| Parents limiting screen-viewing time, n (%) | 2,307 (64.6) | 2,081 (59.9) |
| Internet use time^b^ (minutes/day), median (IQR) | 90 (150) | 90 (150) |

Abbreviations: IQR, interquartile range ; SD, standard deviation ; GDP, gross domestic product

^a^ Excludes participants with overweight or obesity.

^b^ Data collected from secondary school children only.

## Supplementary Table 3. Sociodemographic and health-related characteristics of the sample with matched data at both Waves, primary school in comparison to secondary school students (main analysis)

|  | Primary school students (n=3,085) | Secondary school students (n=3,969) |
| --- | --- | --- |
| Socio-demographic characteristics |  |  |
| Age (years), mean (SD) | 10.1 (0.8) | 14.1 (1.7) |
| Female, n (%) | 1,435 (46.5) | 2,042 (51.4) |
| Urban residence, n (%) | 1,581 (51.2) | 2,821 (71.1) |
| GDP, n (%) |  |  |
| Low | 1,123 (36.4) | 1,238 (31.2) |
| Medium | 1,027 (33.3) | 1,250 (31.5) |
| High | 935 (30.3) | 1,481 (37.3) |
| Normal weight^a^, n (%) | 2,351 (76.2) | 3,335 (84) |
| Primary outcomes |  |  |
| Total sedentary behaviour time (minutes/day), median (IQR) | 270 (200) | 360 (260) |
| Electronic device use time (minutes/day), median (IQR) | 30 (80) | 100 (150) |
| Homework time (hours/day), n (%) |  |  |
| 0 hours | 12 (0.4) | 38 (1) |
| <1 hour | 755 (25.9) | 611 (16.9) |
| 1-2 hours | 1,432 (49.1) | 1,277 (35.2) |
| 2-3 hours | 508 (17.4) | 941 (26) |
| ≥3 hours | 209 (7.2) | 757 (20.9) |
| Out-of-campus learning time (hours/day), n (%) |  |  |
| 0 hours | 1,600 (54.2) | 2691 (71) |
| <1 hour | 199 (6.7) | 285 (7.5) |
| 1-2 hours | 507 (17.2) | 260 (6.9) |
| 2-3 hours | 325 (11) | 202 (5.3) |
| ≥3 hours | 319 (10.8) | 353 (9.3) |
| Secondary outcomes |  |  |
| Screen-viewing time (minutes/day), median (IQR) | 120 (140) | 210 (250) |
| Meeting screen-viewing time recommendation, n (%) | 1,501 (48.9) | 998 (25.2) |
| Meeting regulatory requirement on homework time, n (%) | 767 (26.3) | 1,926 (53.1) |
| Parents limiting screen-viewing time, n (%) | 2,345 (76.2) | 2,043 (51.5) |
| Internet use time^b^ (minutes/day), median (IQR) | NA | 90 (150) |

Abbreviations: IQR, interquartile range; NA, non-applicable ; SD, standard deviation ; GDP, gross domestic product.

^a^ Excludes participants with overweight or obesity.

^b^ Data collected from secondary school children only.

## Supplementary Table 4. Sociodemographic and health-related characteristics of the sample with matched data at both Waves, urban in comparison to rural residents (main analysis)

|  | Urban residents (n=4,402) | Rural residents (n=2,652) |
| --- | --- | --- |
| Socio-demographic characteristics |  |  |
| Age (years), mean (SD) | 12.7 (2.5) | 11.8 (2.1) |
| Female, n (%) | 2,114 (48) | 1,363 (51.4) |
| Secondary school^a^, n (%) | 2,821 (64.1) | 1,148 (43.3) |
| GDP, n (%) |  |  |
| Low | 1,472 (33.4) | 889 (33.5) |
| Medium | 1,424 (32.3) | 853 (32.2) |
| High | 1,506 (34.2) | 910 (34.3) |
| Normal weight^a^, n (%) | 3,401 (77.3) | 2,285 (86.2) |
| Primary outcomes |  |  |
| Total sedentary behaviour time (minutes/day), median (IQR) | 330 (230) | 280 (240) |
| Electronic device use time (minutes/day), median (IQR) | 60 (130) | 60 (123) |
| Homework time (hours/day), n (%) |  |  |
| 0 hours | 27 (0.7) | 23 (1) |
| <1 hour | 691 (16.7) | 675 (28) |
| 1-2 hours | 1,544 (37.4) | 1,165 (48.4) |
| 2-3 hours | 1,108 (26.8) | 341 (14.2) |
| ≥3 hours | 761 (18.4) | 205 (8.5) |
| Out-of-campus learning time (hours/day), n (%) |  |  |
| 0 hours | 2,559 (60.3) | 1,732 (69.3) |
| <1 hour | 259 (6.1) | 225 (9) |
| 1-2 hours | 474 (11.2) | 293 (11.7) |
| 2-3 hours | 393 (9.3) | 134 (5.4) |
| ≥3 hours | 557 (13.1) | 115 (4.6) |
| Secondary outcomes |  |  |
| Screen-viewing time (minutes/day), median (IQR) | 150 (200.5) | 150 (215) |
| Meeting screen-viewing time recommendation, n (%) | 1,548 (35.3) | 951 (36) |
| Meeting regulatory requirement on homework time, n (%) | 1,580 (38.2) | 1,113 (46.2) |
| Parents limiting screen-viewing time, n (%) | 2,799 (63.7) | 1,589 (60.1) |
| Internet use time^b^ (minutes/day), median (IQR) | 90 (150) | 90 (180) |

Abbreviations: IQR, interquartile range; SD, standard deviation; GDP, gross domestic product.

^a^Excludes participants with overweight or obesity.

^b^Data collected from secondary school children only.

## Supplementary Table 5. Sociodemographic and health-related characteristics of the sample with matched data at both Waves, participants with normal weight in comparison to overweigh/obesity at baseline (main analysis).

|  | Normal weight (n=5,686) | Overweight or obesity (n=1,368) |
| --- | --- | --- |
| Socio-demographic characteristics |  |  |
| Age (years), mean (SD) | 12.5 (2.4) | 11.8 (2.3) |
| Female, n (%) | 2,993 (52.6) | 484 (35.4) |
| Secondary school, n (%) | 3,335 (58.7) | 634 (46.3) |
| Urban residence, n (%) | 3,401 (59.8) | 1001 (73.2) |
| GDP, n (%) |  |  |
| Low | 1,921 (33.8) | 440 (32.2) |
| Medium | 1,883 (33.1) | 394 (28.8) |
| High | 1,882 (33.1) | 534 (39) |
| Primary outcomes |  |  |
| Electronic device use time (minutes/day), median (IQR) | 60 (130) | 60 (110) |
| Homework time (hours/day), n (%) |  |  |
| 0 hours | 42 (0.8) | 8 (0.6) |
| <1 hour | 1,123 (21.3) | 243 (19.1) |
| 1-2 hours | 2,207 (41.9) | 502 (39.4) |
| 2-3 hours | 1,131 (21.5) | 318 (25) |
| ≥3 hours | 764 (14.5) | 202 (15.9) |
| Out-of-campus learning time (hours/day), n (%) |  |  |
| 0 hours | 3,561 (65.8) | 730 (55.1) |
| <1 hour | 403 (7.4) | 81 (6.1) |
| 1-2 hours | 556 (10.3) | 211 (15.9) |
| 2-3 hours | 397 (7.3) | 130 (9.8) |
| ≥3 hours | 498 (9.2) | 174 (13.1) |
| Total sedentary behaviour time (minutes/day), median (IQR) | 320 (240) | 330 (230) |
| Secondary outcomes |  |  |
| Screen-viewing time (minutes/day), median (IQR) | 150 (209.5) | 150 (180) |
| Meeting screen-viewing time recommendation, n (%) | 2,002 (35.3) | 497 (36.5) |
| Meeting regulatory requirement on homework time, n (%) | 2,250 (42.7) | 443 (34.8) |
| Parents limiting screen-viewing time, n (%) | 3,454 (60.9) | 934 (68.3) |
| Internet use time^a^ (minutes/day), median (IQR) | 90 (150) | 90 (150) |

Abbreviations: IQR, interquartile range; SD, standard deviation; GDP, gross domestic product

^a^Data collected from secondary school children only.

## Supplementary Table 6. Percentage changes in sedentary behaviours of participants taking part in both waves after the introduction of nationwide regulations, boys in comparison to girls.

|  | Boys | | | | Girls | | | |
| --- | --- | --- | --- | --- | --- | --- | --- | --- |
|  | n | Estimate | 95% CI | *p* value^a^ | n | Estimate | 95% CI | *p* value^a^ |
| Primary outcomes |  |  |  |  |  |  |  |  |
| Total sedentary behaviour time^b^ |  |  |  |  |  |  |  |  |
| Model 1^c^ | 3,159 | -10.4 | (-12.8, -7.9) | <0.001 | 3,027 | -11.6 | (-14.1, -9.1) | <0.001 |
| Model 2^d^ | 3,159 | -14.1 | (-16.9, -11.2) | <0.001 | 3,027 | -14.4 | (-17.4, -11.5) | <0.001 |
| Electronic device use time^e^ |  |  |  |  |  |  |  |  |
| Model 1^c^ | 3,824 | 5.1 | (-0.2, 10.4) | 0.06 | 3,714 | 4.6 | (-0.9, 10.0) | 0.10 |
| Model 2^d^ | 3,824 | -5.3 | (-12·0, 1.4) | 0.12 | 3,714 | -5.1 | (-11.8, 1.6) | 0.14 |
| Secondary outcomes |  |  |  |  |  |  |  |  |
| Screen-viewing time^f^ | |  |  |  |  |  |  |  |
| Model 1^c^ | 3,823 | -0.9 | (-4.5, 2.7) | 0.62 | 3,713 | -4.4 | (-8.0, -0.9) | 0.02 |
| Model 2^d^ | 3,823 | -5.5 | (-9.8, -1.2) | 0.011 | 3,713 | -8.5 | (-12.8, -4.2) | <0.001 |
| Internet use time^g^ | |  |  |  |  |  |  |  |
| Model 1^c^ | 2,046 | -3.4 | (-11.0, 4.3) | 0.39 | 2,160 | 4.2 | (-3.0, 11.4) | 0.25 |
| Model 2^d^ | 2,046 | -7.5 | (-16.7, 1.7) | 0.11 | 2,160 | 1.7 | (-6.8, 10.2) | 0.70 |

Abbreviations: CI, confidence interval.

^a^Significance assessed at *p* < 0.005 using the Bonferroni correction.

^b^Calculated in minutes as the sum of self-reported electronic device use time per day, TV/video game use time per day, computer use time per day, homework time, and out-of-campus learning time.

^c^Unadjusted model.

^d^Model 1 + age, sex, socioeconomic status, and area of residence.

^e^Calculated in minutes from self-reported average time (hours and minutes) per day spent using mobile phones, handheld game consoles, and tablets.

^f^Calculated in minutes as the sum of self-reported electronic device use time per day, TV/video game use time per day, and computer use time per day.

^g^Calculated in minutes from self-reported average time (hours and minutes) per day spent ‘online’; only measured in secondary school children.

## Supplementary Table 7. Odds ratio for being one category up in homework and out-of-campus learning time, and for meeting recommendations, in participants taking part in both waves after the introduction of nationwide regulations, boys in comparison to girls (main analysis).

|  | Boys | | | | Girls | | | |
| --- | --- | --- | --- | --- | --- | --- | --- | --- |
|  | n | Estimate | 95% CI | *p* value^a^ | n | Estimate | 95% CI | *p* value^a^ |
| Primary outcomes |  |  |  |  |  |  |  |  |
| Homework time ^b, c^ |  |  |  |  |  |  |  |  |
| Model 1^d^ | 2,711 | 0.41 | (0.37, 0.46) | <0.001 | 2,436 | 0.43 | (0.39, 0.49) | <0.001 |
| Model 2^e^ | 2,711 | 0.37 | (0.32, 0.42) | <0.001 | 2,436 | 0.38 | (0.33, 0.43) | <0.001 |
| Out-of-campus learning time^b, f^ |  |  |  |  |  |  |  |  |
| Model 1^d^ | 2,753 | 0.51 | (0.45, 0.59) | <0.001 | 2,606 | 0.51 | (0.45, 0.59) | <0.001 |
| Model 2^e^ | 2,753 | 0.53 | (0.45, 0.62) | <0.001 | 2,606 | 0.54 | (0.46, 0.64) | <0.001 |
| Secondary outcomes |  |  |  |  |  |  |  |  |
| Meeting screen-viewing time recommendations ^g, h^ | |  |  |  |  |  |  |  |
| Model 1^d^ | 3,823 | 1.02 | (0.91, 1.14) | 0.75 | 3,713 | 1.12 | (1.00, 1.26) | 0.054 |
| Model 2^e^ | 3,823 | 1.11 | (0.98, 1.27) | 0.11 | 3,713 | 1.32 | (1.16, 1.51) | <0.001 |
| Meeting regulatory requirement on homework time ^c, g^ | |  |  |  |  |  |  |  |
| Model 1^d^ | 2,711 | 2.76 | (2.40, 3.17) | <0.001 | 2,436 | 2.62 | (2.26, 3.03) | <0.001 |
| Model 2^e^ | 2,711 | 2.73 | (2.31, 3.22) | <0.001 | 2,436 | 2.63 | (2.22, 3.12) | <0.001 |

Abbreviations: CI, confidence interval.

^a^Significance assessed at *p* < 0.005 using the Bonferroni correction.

^b^Odds ratio for being one category up of time spent doing homework/out-of-campus learning (i.e., spending more time on these activities).

^c^Homework time calculated from self-reported categories of time spent doing homework.

^d^Unadjusted model.

^e^Model 1 + age, sex, GDP, and area of residence.

^f^Calculated in self-reported categories of time spent in tutorial classes like English, math, and writing.

^g^Odds ratio for meeting screen-viewing recommendations and homework time regulatory requirements.

^h^Screen time calculated as the sum of self-reported electronic device use time per day, TV/video game use time per day, and computer use time per day.

## Supplementary Table 8. Percentage changes in sedentary behaviours of participants taking part in both waves after the introduction of nationwide regulations, primary in comparison to secondary school students (main analysis).

|  | Primary school students | | | | Secondary school students | | | |
| --- | --- | --- | --- | --- | --- | --- | --- | --- |
|  | n | Estimate | 95% CI | *p* value^a^ | n | Estimate | 95% CI | *p* value^a^ |
| Primary outcomes |  |  |  |  |  |  |  |  |
| Total sedentary behaviour time^b^ |  |  |  |  |  |  |  |  |
| Model 1^c^ | 2,596 | -13.0 | (-15.8, -10.2) | <0.001 | 3,363 | -10.2 | (-12.5, -7.8) | <0.001 |
| Model 2^d^ | 2,596 | -19.1 | (-22.7, -15.5) | <0.001 | 3,363 | -9.5 | (-12.2, -6.8) | <0.001 |
| Electronic device use time^e^ |  |  |  |  |  |  |  |  |
| Model 1^c^ | 3,060 | 18.7 | (11.3, 26.2) | <0.001 | 4,185 | -1.2 | (-6.0, 3.7) | 0.64 |
| Model 2^d^ | 3,060 | 0.8 | (-8.6, 10.3) | 0.86 | 4,185 | -3.6 | (-9.3, 2.1) | 0.22 |
| Secondary outcomes |  |  |  |  |  |  |  |  |
| Screen-viewing time^f^ | |  |  |  |  |  |  |  |
| Model 1^c^ | 3,057 | 7.8 | (3.8, 11.8) | <0.001 | 4,187 | -9.1 | (-12.4, -5.7) | <0.001 |
| Model 2^d^ | 3,057 | -2.3 | (-7.6, 2.9) | 0.38 | 4,187 | -8.4 | (-12.4, -4.3) | <0.001 |
| Internet use time^g^ | |  |  |  |  |  |  |  |
| Model 1^c^ | NA | NA | NA | NA | NA | NA | NA | NA |
| Model 2^d^ | NA | NA | NA | NA | NA | NA | NA | NA |

Abbreviations: CI, confidence interval; NA, non-applicable.

^a^Significance assessed at *p* < 0.005 using the Bonferroni correction.

^b^Calculated in minutes as the sum of self-reported electronic device use time per day, TV/video game use time per day, computer use time per day, homework time, and out-of-campus learning time.

^c^Unadjusted model.

^d^Model 1 + age, sex, socioeconomic status, and area of residence.

^e^Calculated in minutes from self-reported average time (hours and minutes) per day spent using mobile phones, handheld game consoles, and tablets.

^f^Calculated in minutes as the sum of self-reported electronic device use time per day, TV/video game use time per day, and computer use time per day.

^g^Calculated in minutes from self-reported average time (hours and minutes) per day spent ‘online’; only measured in secondary school children.

## Supplementary Table 9. Odds ratio for being one category up in homework and out-of-campus learning time, and for meeting recommendations taking part in both waves after the introduction of nationwide regulations, primary in comparison to secondary school students (main analysis).

|  | Primary school students | | | | Secondary school students | | | |
| --- | --- | --- | --- | --- | --- | --- | --- | --- |
|  | n | Estimate | 95% CI | *p* value^a^ | n | Estimate | 95% CI | *p* value^a^ |
| Primary outcomes |  |  |  |  |  |  |  |  |
| Homework time^b, c^ |  |  |  |  |  |  |  |  |
| Model 1^d^ | 2,801 | 0.32 | (0.28, 0.35) | <0.001 | 2,156 | 0.60 | (0.53, 0.68) | <0.001 |
| Model 2^e^ | 2,801 | 0.30 | (0.26, 0.34) | <0.001 | 2,156 | 0.58 | (0.50, 0.67) | <0.001 |
| Out-of-campus learning time^b, f^ |  |  |  |  |  |  |  |  |
| Model 1^d^ | 2,848 | 0.47 | (0.41, 0.52) | <0.001 | 2,311 | 0.61 | (0.52, 0.70) | <0.001 |
| Model 2^e^ | 2,848 | 0.47 | (0.40, 0.55) | <0.001 | 2,311 | 0.59 | (0.49, 0.71) | <0.001 |
| Secondary outcomes |  |  |  |  |  |  |  |  |
| Meeting screen-viewing time recommendations^g, h^ | |  |  |  |  |  |  |  |
| Model 1^d^ | 3,057 | 0.79 | (0.70, 0.89) | <0.001 | 4,187 | 1.45 | (1.29, 1.62) | <0.001 |
| Model 2^e^ | 3,057 | 1.02 | (0.88, 1.18) | 0.81 | 4,187 | 1.41 | (1.23, 1.61) | <0.001 |
| Meeting regulatory requirement on homework time ^c, g^ | |  |  |  |  |  |  |  |
| Model 1^d^ | 2,801 | 3.24 | (2.84, 3.69) | <0.001 | 2,156 | 1.94 | (1.67, 2.27) | <0.001 |
| Model 2^e^ | 2,801 | 3.61 | (3.09, 4.22) | <0.001 | 2,156 | 2.11 | (1.74, 2.56) | <0.001 |

Abbreviations: CI, confidence interval.

^a^Significance assessed at *p* < 0.005 using the Bonferroni correction.

^b^Odds ratio for being one category up of time spent doing homework/out-of-campus learning (i.e., spending more time on these activities).

^c^Homework time calculated from self-reported categories of time spent doing homework.

^d^Unadjusted model.

^e^Model 1 + age, sex, GDP, and area of residence.

^f^Calculated in self-reported categories of time spent in tutorial classes like English, math, and writing.

^g^Odds ratio for meeting screen-viewing recommendations and homework time regulatory requirements.

^h^Screen time calculated as the sum of self-reported electronic device use time per day, TV/video game use time per day, and computer use time per day.

## Supplementary Table 10. Percentage changes in sedentary behaviours of participants taking part in both waves after the introduction of nationwide regulations, urban in comparison to rural residents (main analysis).

|  | Urban residents | | | | Rural residents | | | |
| --- | --- | --- | --- | --- | --- | --- | --- | --- |
|  | n | Estimate | 95% CI | *p* value^a^ | n | Estimate | 95% CI | *p* value^a^ |
| Primary outcomes |  |  |  |  |  |  |  |  |
| Total sedentary behaviour time^b^ |  |  |  |  |  |  |  |  |
| Model 1^c^ | 3,777 | -12.7 | (-14.8, -10.6) | <0.001 | 2,182 | -7.4 | (-10.4, -4.3) | <0.001 |
| Model 2^d^ | 3,777 | -15.3 | (-17.8, -12.7) | <0.001 | 2,182 | -11.2 | (-15.0, -7.4) | <0.001 |
| Electronic device use time^e^ |  |  |  |  |  |  |  |  |
| Model 1^c^ | 4,486 | 1.7 | (-2.7, 6.2) | 0.45 | 2,759 | 10.7 | (3.7, 17.6) | 0.0026 |
| Model 2^d^ | 4,486 | -3.4 | (-8.9, 2.1) | 0.22 | 2,759 | -3.5 | (-12.7, 5.7) | 0.45 |
| Secondary outcomes |  |  |  |  |  |  |  |  |
| Screen-viewing time^f^ | |  |  |  |  |  |  |  |
| Model 1^c^ | 4,485 | -4.4 | (-7.6, -1.3) | 0.0057 | 2,759 | 0.7 | (-3.5, 4.9) | 0.74 |
| Model 2^d^ | 4,485 | -6.6 | (-10.5, -2.8) | <0.001 | 2,759 | -6.2 | (-11.5, -0.8) | 0.02 |
| Internet use time^g^ | |  |  |  |  |  |  |  |
| Model 1^c^ | 2,872 | -3.2 | (-9.2, 2.7) | 0.29 | 1,184 | 10.3 | (-0.2, 20.7) | 0.053 |
| Model 2^d^ | 2,872 | -6.5 | (-13.7, 0.7) | 0.076 | 1,184 | 7.0 | (-5.4, 19.3) | 0.27 |

Abbreviations: CI, confidence interval.

^a^ Significance assessed at *p* < 0.005 using the Bonferroni correction.

^b^ Calculated in minutes as the sum of self-reported electronic device use time per day, TV/video game use time per day, computer use time per day, homework time, and out-of-campus learning time.

^c^ Unadjusted model.

^d^ Model 1 + age, sex, socioeconomic status, and area of residence.

^e^ Calculated in minutes from self-reported average time (hours and minutes) per day spent using mobile phones, handheld game consoles, and tablets.

^f^ Calculated in minutes as the sum of self-reported electronic device use time per day, TV/video game use time per day, and computer use time per day.

^g^ Calculated in minutes from self-reported average time (hours and minutes) per day spent ‘online’; only measured in secondary school children.

## Supplementary Table 11. Odds ratio for being one category up in homework and out-of-campus learning time, and for meeting recommendations screen-viewing time in participants taking part in both waves after the introduction of nationwide regulations, urban in comparison to rural residents (main analysis).

|  | Urban residents | | | | Rural residents | | | |
| --- | --- | --- | --- | --- | --- | --- | --- | --- |
|  | n | Estimate | 95% CI | *p* value^a^ | n | Estimate | 95% CI | *p* value^a^ |
| Primary outcomes |  |  |  |  |  |  |  |  |
| Homework time^b, c^ |  |  |  |  |  |  |  |  |
| Model 1^d^ | 2,910 | 0.39 | (0.35, 0.43) | <0.001 | 2,047 | 0.48 | (0.42, 0.54) | <0.001 |
| Model 2^e^ | 2,910 | 0.34 | (0.30, 0.39) | <0.001 | 2,047 | 0.45 | (0.39, 0.52) | <0.001 |
| Out-of-campus learning time^b, f^ |  |  |  |  |  |  |  |  |
| Model 1^d^ | 2,984 | 0.50 | (0.45, 0.57) | <0.001 | 2,175 | 0.55 | (0.47, 0.65) | <0.001 |
| Model 2^e^ | 2,984 | 0.47 | (0.41, 0.55) | <0.001 | 2,175 | 0.65 | (0.54, 0.79) | <0.001 |
| Secondary outcomes |  |  |  |  |  |  |  |  |
| Meeting screen-viewing time recommendations^g, h^ | |  |  |  |  |  |  |  |
| Model 1^d^ | 4,485 | 1.16 | (1.04, 1.29) | 0.0065 | 2,759 | 0.95 | (0.83, 1.08) | 0.40 |
| Model 2^e^ | 4,485 | 1.29 | (1.14, 1.46) | <0.001 | 2,759 | 1.07 | (0.92, 1.24) | 0.40 |
| Meeting regulatory requirement on homework time^c, g^ | |  |  |  |  |  |  |  |
| Model 1^d^ | 2,910 | 2.79 | (2.45, 3.18) | <0.001 | 2,047 | 2.49 | (2.13, 2.91) | <0.001 |
| Model 2^e^ | 2,910 | 3.04 | (2.60, 3.56) | <0.001 | 2,047 | 2.46 | (2.05, 2.96) | <0.001 |

Abbreviations: CI, confidence interval.

^a^Significance assessed at *p* < 0.005 using the Bonferroni correction.

^b^Odds ratio for being one category up of time spent doing homework/out-of-campus learning (i.e., spending more time on these activities).

^c^Homework time calculated from self-reported categories of time spent doing homework.

^d^Unadjusted model.

^e^Model 1 + age, sex, GDP, and area of residence.

^f^Calculated in self-reported categories of time spent in tutorial classes like English, math, and writing

^g^Odds ratio for meeting screen-viewing recommendations and homework time regulatory requirements.

^h^Screen time calculated as the sum of self-reported electronic device use time per day, TV/video game use time per day, and computer use time per day.

## Supplementary Table 12. Percentage changes in sedentary behaviours of participants taking part in both waves after the introduction of nationwide regulations, participant with normal weight in comparison to participants who have overweight/obesity (main analysis).

|  | Normal weight | | | | Overweight or obesity | | | |
| --- | --- | --- | --- | --- | --- | --- | --- | --- |
|  | n | Estimate | 95% CI | *p* value^a^ | n | Estimate | 95% CI | *p* value^a^ |
| Primary outcomes |  |  |  |  |  |  |  |  |
| Total sedentary behaviour time^b^ |  |  |  |  |  |  |  |  |
| Model 1^c^ | 4,783 | -10.1 | (-12.1, -8.1) | <0.001 | 1,176 | -14.0 | (-17.8, -10.2) | <0.001 |
| Model 2^d^ | 4,783 | -13.2 | (-15.5, -10.8) | <0.001 | 1,176 | -18.1 | (-22.5, -13.8) | <0.001 |
| Electronic device use time^e^ |  |  |  |  |  |  |  |  |
| Model 1^c^ | 5,853 | 5.4 | (1.1, 9.6) | 0.013 | 1,392 | 3.3 | (-5.2, 11.9) | 0.44 |
| Model 2^d^ | 5,853 | -3.7 | (-9, 1.6) | 0.17 | 1,392 | -9.4 | (-20.1, 1.3) | 0.086 |
| Secondary outcomes |  |  |  |  |  |  |  |  |
| Screen-viewing time^f^ |  |  |  |  |  |  |  |  |
| Model 1^c^ | 5,853 | -2.6 | (-5.4, 0.2) | 0.071 | 1,391 | -2.3 | (-7.9, 3.4) | 0.44 |
| Model 2^d^ | 5,853 | -6.7 | (-10.2, -3.3) | <0.001 | 1,391 | -7.4 | (-14.2, -0.7) | 0.03 |
| Internet use time^g^ | |  |  |  |  |  |  |  |
| Model 1^c^ | 3,414 | 0.7 | (-5.0, 6.4) | 0.81 | 642 | 1.2 | (-11.6, 14.0) | 0.86 |
| Model 2^d^ | 3,414 | -4.0 | (-10.9, 2.8) | 0.25 | 642 | 5.1 | (-9.6, 19.7) | 0.50 |

Abbreviations: CI, confidence interval.

^a^ Significance assessed at *p* < 0.005 using the Bonferroni correction.

^b^ Calculated in minutes as the sum of self-reported electronic device use time per day, TV/video game use time per day, computer use time per day, homework time, and out-of-campus learning time.

^c^ Unadjusted model.

^d^ Model 1 + age, sex, socioeconomic status, and area of residence.

^e^ Calculated in minutes from self-reported average time (hours and minutes) per day spent using mobile phones, handheld game consoles, and tablets.

^f^ Calculated in minutes as the sum of self-reported electronic device use time per day, TV/video game use time per day, and computer use time per day.

^g^ Calculated in minutes from self-reported average time (hours and minutes) per day spent ‘online’; only measured in secondary school children.

## Supplementary Table 13. Odds ratio for being one category up in homework and out-of-campus learning time, and for meeting recommendations in participants taking part in both waves after the introduction of nationwide regulations, participant with normal weight in comparison to participants who have overweight/obesity (main analysis).

|  | Normal weight | | | | Overweight or obesity | | | | |
| --- | --- | --- | --- | --- | --- | --- | --- | --- | --- |
|  | n | Estimate | 95% CI | *p* value^a^ | n | Estimate | | 95% CI | *p* value^a^ |
| Primary outcomes |  |  |  |  |  |  | |  |  |
| Homework time^b, c^ |  |  |  |  |  |  | |  |  |
| Model 1^d^ | 3,914 | 0.43 | (0.39, 0.47) | <0.001 | 1,043 | 0.40 | | (0.33, 0.47) | <0.001 |
| Model 2^e^ | 3,914 | 0.39 | (0.35, 0.44) | <0.001 | 1,043 | 0.31 | | (0.25, 0.39) | <0.001 |
| Out-of-campus learning time^b, f^ |  |  |  |  |  | |  |  |  |
| Model 1^d^ | 4,078 | 0.56 | (0.50, 0.62) | <0.001 | 1,081 | 0.40 | | (0.32, 0.49) | <0.001 |
| Model 2^e^ | 4,078 | 0.58 | (0.51, 0.66) | <0.001 | 1,081 | 0.41 | | (0.32, 0.53) | <0.001 |
| Secondary outcomes |  |  |  |  |  |  | |  |  |
| Meeting screen-viewing time recommendations^g, h^ | |  |  |  |  |  | |  |  |
| Model 1^d^ | 5,853 | 1.10 | (1.00, 1.20) | 0.050 | 1,391 | 0.97 | | (0.81, 1.16) | 0.73 |
| Model 2^e^ | 5,853 | 1.24 | (1.12, 1.38) | <0.001 | 1,391 | 1.08 | | (0.89, 1.32) | 0.44 |
| Meeting regulatory requirement on homework time ^c, g^ | |  |  |  |  |  | |  |  |
| Model 1^d^ | 3,914 | 2.64 | (2.36, 2.96) | <0.001 | 1,043 | 2.76 | | (2.22, 3.43) | <0.001 |
| Model 2^e^ | 3,914 | 2.70 | (2.37, 3.08) | <0.001 | 1,043 | 2.63 | | (2.03, 3.40) | <0.001 |

Abbreviations: CI, confidence interval.

^a^ Significance assessed at *p* < 0.005 using the Bonferroni correction.

^b^ Odds ratio for being one category up of time spent doing homework/out-of-campus learning (i.e., spending more time on these activities).

^c^ Homework time calculated from self-reported categories of time spent doing homework.

^d^ Unadjusted model.

^e^ Model 1 + age, sex, GDP, and area of residence.

^f^ Calculated in self-reported categories of time spent in tutorial classes like English, math, and writing.

^g^ Odds ratio for meeting screen-viewing recommendations and homework time regulatory requirements.

^h^ Screen time calculated as the sum of self-reported electronic device use time per day, TV/video game use time per day, and computer use time per day.

# Results of exploratory analysis based on full sample (repeated cross-sectional analyses using representative samples)

## Supplementary Table 14. Sociodemographic and health-related characteristics of the complete sample by periods of the introduction of nationwide regulatory interventions (exploratory repeated cross-sectional analyses)

|  | Wave 1 (before regulations) (n=46,485) | Wave 2 (after introduction of regulations) (n=45,738) |
| --- | --- | --- |
| Socio-demographic characteristics |  |  |
| Age (years), mean (SD) | 13.3 (2.6) | 13.4 (2.6) |
| Female, n (%) | 23,112 (49.7) | 22,535 (49.3) |
| Secondary school, n (%) | 30,669 (65.9) | 30,091 (65.7) |
| Urban, n (%) | 26,860 (57.8) | 28,928 (63.2) |
| GDP n (%) |  |  |
| Low | 15,858 (34.1) | 15,162 (33.1) |
| Medium | 16,297 (35.1) | 15,405 (33.7) |
| High | 14,330 (30.8) | 15,171 (33.2) |
| Normal weight^a^ n (%) | 37,890 (81.5) | 36,856 (80.8) |
| Primary outcomes |  |  |
| Total sedentary behaviour time (minutes/day), median (IQR) | 330 (260) | 300 (265) |
| Electronic device use time (minutes/day), median (IQR) | 60 (140) | 65 (168) |
| Homework time (hours/day), n (%) |  |  |
| 0 hours | 477 (1.1) | 783 (1.8) |
| <1 hour | 9,329 (21.7) | 13,068 (30.7) |
| 1-2 hours | 16,598 (38.6) | 16,229 (38.2) |
| 2-3 hours | 9,095 (21.1) | 6,897 (16.2) |
| ≥3 hours | 7,537 (17.5) | 5,556 (13.1) |
| Out-of-campus learning time (hours/day), n (%) |  |  |
| 0 hours | 27,767 (62.4) | 32,448 (74.2) |
| <1 hour | 4,079 (9.2) | 3,270 (7.5) |
| 1-2 hours | 4,944 (11.1) | 4,516 (10.3) |
| 2-3 hours | 3,399 (7.6) | 1,822 (4.2) |
| ≥3 hours | 4,292 (9.6) | 1,686 (3.9) |
| Secondary outcomes |  |  |
| Total screen viewing time (minutes/day), median (IQR) | 160 (230) | 180 (220) |
| Internet use time (minutes/day), median (IQR) | 90 (157) | 90 (165) |
| Meeting screen-viewing time recommendation, n (%) | 16,587 (35.9) | 15,863 (34.9) |
| Meeting regulatory requirement on homework time, n (%) | 19,121 (44.4) | 24,195 (56.9) |

Abbreviations: IQR, interquartile range; SD, standard deviation; GDP, gross domestic product.

^a^Excludes participants with overweight or obesity.

## Supplementary Table 15. Sociodemographic and health-related characteristics of the complete sample by periods of the introduction of nationwide regulatory interventions, boys in comparison to girls (exploratory repeated cross-sectional analyses).

|  | **Boys** |  | **Girls** |  |
| --- | --- | --- | --- | --- |
|  | Wave 1 (before regulations)  (n= 23,373) | Wave 2 (after introduction of regulations)  (n= 23,203) | Wave 1 (before regulations)  (n= 23,112) | Wave 2 (after introduction of regulations)  (n= 22,535) |
| **Socio-demographic characteristics** |  |  |  |  |
| Age (years), mean (SD) | 13.3 (2.6) | 13.3 (2.6) | 13.4 (2.6) | 13.4 (2.6) |
| Secondary school, n (%) | 14,881 (63.7) | 14,844 (63.9) | 15,788 (68.3) | 15,247 (67.7) |
| Urban residence, n (%) | 13,620 (58.3) | 14,868 (64.1) | 13240 (57.3) | 14,060 (62.4) |
| GDP, n (%) |  |  |  |  |
| Low | 7,892 (33.8) | 7,764 (33.5) | 7,966 (34.5) | 7,398 (32.8) |
| Medium | 8,033 (34.4) | 7,512 (32.4) | 8,264 (35.8) | 7,893 (35) |
| High | 7,448 (31.9) | 7,927 (34.2) | 6,882 (29.8) | 7,244 (32.1) |
| Normal weight ^a^, n (%) | 18,028 (77.2) | 17,639 (76.2) | 19,862 (86) | 19,217 (85.5) |
| **Primary outcomes** |  |  |  |  |
| Total sedentary behaviour time (minutes/day), median (IQR) | 330 (270) | 300 (270) | 330 (240) | 300 (245) |
| Electronic device use time (minutes/day), median (IQR) | 60 (140) | 64 (170) | 60 (140) | 65 (155) |
| Homework time (hours/day), n (%) |  |  |  |  |
| 0 hours | 295 (1.4) | 521 (2.4) | 182 (0.9) | 262 (1.3) |
| <1 hour | 5,077 (23.3) | 7,132 (32.9) | 4,252 (20) | 5,936 (28.4) |
| 1-2 hours | 8,614 (39.5) | 8,307 (38.4) | 7,984 (37.6) | 7,922 (37.9) |
| 2-3 hours | 4,429 (20.3) | 3,227 (14.9) | 4,666 (22) | 3,670 (17.6) |
| ≥3 hours | 3,383 (15.5) | 2,468 (11.4) | 4,154 (19.6) | 3,088 (14.8) |
| Out-of-campus learning time (hours/day), n (%) |  |  |  |  |
| 0 hours | 13,405 (60.2) | 16,223 (73.4) | 14,362 (64.7) | 16,225 (75) |
| <1 hour | 2,340 (10.5) | 1,829 (8.3) | 1,739 (7.8) | 1,441 (6.7) |
| 1-2 hours | 2,588 (11.6) | 2,311 (10.5) | 23,56 (10.6) | 2,205 (10.2) |
| 2-3 hours | 1,723 (7.7) | 857 (3.9) | 1,676 (7.5) | 965 (4.5) |
| ≥3 hours | 2,211 (9.9) | 893 (4) | 2,081 (9.4) | 793 (3.7) |
| **Secondary outcomes** |  |  |  |  |
| Meeting regulatory requirement on homework time, n (%) | 180 (215) | 180 (240) | 150 (240) | 150 (230) |
| Internet use time (minutes/day), median (IQR) | 90 (150) | 90 (165) | 83 (160) | 90 (165) |
| Meeting screen-viewing time recommendations, n (%) | 7,843 (33.8) | 7,569 (32.8) | 8,744 (38.1) | 8,294 (37) |
| Screen-viewing time (minutes/day), median (IQR) | 10,058 (46.1) | 12,833 (59.3) | 9,063 (42.7) | 11,362 (54.4) |

Abbreviations: IQR, interquartile range; SD, standard deviation; GDP, gross domestic product

^a^Excludes participants with overweight or obesity.

## Supplementary Table 16. Sociodemographic and health-related characteristics of the complete sample by periods of the introduction of nationwide regulatory interventions, primary school in comparison to secondary school students (exploratory repeated cross-sectional analyses).

|  | Primary school students | | Secondary school students | |
| --- | --- | --- | --- | --- |
|  | Wave 1 (before regulations) (n= 15,816) | Wave 2 (after introduction of regulations) (n= 15,647) | Wave 1 (before regulations) (n= 30,669) | Wave 2 (after introduction of regulations) (n= 30,091) |
| Socio-demographic characteristics |  |  |  |  |
| Age (years), mean (SD) | 10.5 (1) | 10.4 (1) | 14.8 (1.7) | 14.9 (1.8) |
| Female, n (%) | 7,324 (46.3) | 7,288 (46.6) | 15,788 (51.5) | 15,247 (50.7) |
| Urban residence, n (%) | 7,722 (48.8) | 8,686 (55.5) | 19,138 (62.4) | 20,242 (67.3) |
| GDP, n (%) |  |  |  |  |
| Low | 5,784 (36.6) | 5,288 (33.8) | 10,074 (32.8) | 9,874 (32.8) |
| Medium | 5,285 (33.4) | 5,247 (33.5) | 11,012 (35.9) | 10,158 (33.8) |
| High | 4,747 (30) | 5,112 (32.7) | 9,583 (31.2) | 10,059 (33.4) |
| Normal weight^a^, n (%) | 12,018 (76) | 11,835 (75.9) | 25,872 (84.4) | 25,021 (83.3) |
| Primary outcomes |  |  |  |  |
| Total sedentary behaviour time (minutes/day), median (IQR) | 280 (210) | 210 (200) | 360 (270) | 330 (252) |
| Electronic device use time (minutes/day), median (IQR) | 30 (80) | 30 (75) | 90 (165) | 120 (180) |
| Homework time (hours/day), n (%) |  |  |  |  |
| 0 hours | 53 (0.4) | 258 (1.7) | 424 (1.5) | 525 (1.9) |
| <1 hour | 4,002 (26.6) | 7,177 (47.9) | 5,327 (19) | 5,891 (21.4) |
| 1-2 hours | 7,283 (48.4) | 5,885 (39.3) | 9,315 (33.3) | 10,344 (37.6) |
| 2-3 hours | 2,676 (17.8) | 1,212 (8.1) | 6,419 (22.9) | 5,685 (20.6) |
| ≥3 hours | 1,036 (6.9) | 455 (3) | 6,501 (23.2) | 5,101 (18.5) |
| Out-of-campus learning time (hours/day), n (%) |  |  |  |  |
| 0 hours | 7,482 (49.1) | 9,533 (63.3) | 20,285 (69.4) | 22,915 (79.9) |
| <1 hour | 1,045 (6.9) | 1,132 (7.5) | 3,034 (10.4) | 2,138 (7.5) |
| 1-2 hours | 2,855 (18.7) | 2,742 (18.2) | 2,089 (7.1) | 1,774 (6.2) |
| 2-3 hours | 1,906 (12.5) | 951 (6.3) | 1,493 (5.1) | 871 (3) |
| ≥3 hours | 1,948 (12.8) | 691 (4.6) | 2,344 (8) | 995 (3.5) |
| Secondary outcomes |  |  |  |  |
| Screen-viewing time (minutes/day), median (IQR) | 120 (150) | 120 (150) | 210 (270) | 210 (270) |
| Internet use time (minutes/day), median (IQR) | NA | NA | 90 (157) | 90 (165) |
| Meeting screen-viewing time recommendation, n (%) | 7,430 (47.3) | 7,742 (49.8) | 9,157 (30) | 8,121 (27.1) |
| Meeting regulatory requirement on homework time, n (%) | 4,055 (26.9) | 7,435 (49.6) | 15,066 (53.8) | 16,760 (60.8) |

Abbreviations: IQR, interquartile range; SD, standard deviation; GDP, gross domestic product

^a^Excludes participants with overweight or obesity.

## Supplementary Table 17. Sociodemographic and health-related characteristics of the complete sample by periods of the introduction of nationwide regulatory interventions, urban in comparison to rural residents (exploratory repeated cross-sectional analyses).

|  | Urban residents | | Rural residents | |
| --- | --- | --- | --- | --- |
|  | Wave 1 (before regulations) (n= 26,860) | Wave 2 (after introduction of regulations) (n= 28,928) | Wave 1 (before regulations) (n= 19,625) | Wave 2 (after introduction of regulations) (n= 16,810) |
| Socio-demographic characteristics |  |  |  |  |
| Age (years), mean (SD) | 13.7 (2.6) | 13.6 (2.7) | 12.9 (2.4) | 12.9 (2.4) |
| Female, n (%) | 13,240 (49.3) | 14,060 (48.6) | 9,872 (50.3) | 8,475 (50.4) |
| Secondary school, n (%) | 19,138 (71.3) | 20,242 (69.9) | 11,531 (58.8) | 9,849 (58.6) |
| GDP, n (%) |  |  |  |  |
| Low | 9,147 (34.1) | 8,825 (30.5) | 6,711 (34.2) | 6,337 (37.7) |
| Medium | 8,900 (33.1) | 10,161 (35.1) | 7,397 (37.7) | 5,244 (31.2) |
| High | 8,813 (32.8) | 9,942 (34.4) | 5,517 (28.1) | 5,229 (31.1) |
| Normal weight^a^, n (%) | 21,325 (79.4) | 2,2664 (78.5) | 16,565 (84.5) | 14,192 (84.5) |
| Primary outcomes |  |  |  |  |
| Total sedentary behaviour time (minutes/day), median (IQR) | 345 (240) | 300 (255) | 300 (253.5) | 270 (240) |
| Electronic device use time (minutes/day), median (IQR) | 70 (160) | 70 (160) | 60 (130) | 60 (150) |
| Homework time (hours/day), n (%) |  |  |  |  |
| 0 hours | 293 (1.2) | 488 (1.8) | 184 (1) | 295 (1.9) |
| <1 hour | 4,969 (19.9) | 7,521 (27.8) | 4,360 (24.2) | 5,547 (35.8) |
| 1-2 hours | 9,279 (37.1) | 10,272 (38) | 7,319 (40.6) | 5,957 (38.4) |
| 2-3 hours | 5,760 (23) | 4,803 (17.8) | 3,335 (18.5) | 2,094 (13.5) |
| ≥3 hours | 4,720 (18.9) | 3,956 (14.6) | 2,817 (15.6) | 1,600 (10.3) |
| Out-of-campus learning time (hours/day), n (%) |  |  |  |  |
| 0 hours | 15,486 (60) | 19,906 (71.5) | 12,281 (65.8) | 12,542 (78.8) |
| <1 hour | 2,249 (8.7) | 2,227 (8) | 1,830 (9.8) | 1,043 (6.6) |
| 1-2 hours | 2,959 (11.5) | 3,091 (11.1) | 1,985 (10.6) | 1,425 (9) |
| 2-3 hours | 2,243 (8.7) | 1,343 (4.8) | 1,156 (6.2) | 479 (3) |
| ≥3 hours | 2,880 (11.2) | 1,259 (4.5) | 1,412 (7.6) | 427 (2.7) |
| Secondary outcomes |  |  |  |  |
| Screen-viewing time (minutes/day), median (IQR) | 180 (230) | 180 (228) | 150 (215) | 170 (220) |
| Internet use time (minutes/day), median (IQR) | 90 (150) | 90 (160) | 70 (170) | 90 (180) |
| Meeting screen-viewing time recommendation, n (%) | 8,972 (33.5) | 9,959 (34.5) | 7,615 (39.2) | 5,904 (35.4) |
| Meeting regulatory requirement on homework time, n (%) | 11,080 (44.3) | 14,874 (55) | 8,041 (44.6) | 9,321 (60.2) |

Abbreviations: IQR, interquartile range; SD, standard deviation; GDP, gross domestic product

^a^Excludes participants with overweight or obesity

## Supplementary Table 18. Sociodemographic and health-related characteristics of the complete sample by periods of the introduction of nationwide regulatory interventions, participants with normal weight in comparison to overweigh/obesity (exploratory repeated cross-sectional analyses).

|  | Normal weight | | Overweight or obesity | |
| --- | --- | --- | --- | --- |
|  | Wave 1 (before regulations) (n= 37,890) | Wave 2 (after introduction of regulations) (n= 36,856) | Wave 1 (before regulations) (n= 8,581) | Wave 2 (after introduction of regulations) (n= 8,785) |
| Socio-demographic characteristics |  |  |  |  |
| Age (years), mean (SD) | 13.5 (2.6) | 13.5 (2.6) | 12.8 (2.5) | 12.8 (2.6) |
| Female, n (%) | 19,862 (52.4) | 19,217 (52.1) | 3,245 (37.8) | 3,267 (37.2) |
| Secondary, n (%) | 25,872 (68.3) | 25,054 (67.9) | 4,797 (55.9) | 5,037 (57.3) |
| Urban residence, n (%) | 21,325 (56.3) | 22,664 (61.5) | 5,531 (64.5) | 6,191 (70.5) |
| GDP, n (%) |  |  |  |  |
| Low | 13,117 (34.6) | 12,445 (33.8) | 2,740 (31.9) | 2,700 (30.7) |
| Medium | 13,371 (35.3) | 12,478 (33.9) | 2,921 (34) | 2,861 (32.6) |
| High | 11,402 (30.1) | 11,933 (32.4) | 2,920 (34) | 3,224 (36.7) |
| Primary outcomes |  |  |  |  |
| Total sedentary behaviour time (minutes/day), median (IQR) | 330 (250) | 300 (270) | 340 (240) | 300 (260) |
| Electronic device use time (minutes/day), median (IQR) | 60 (140) | 70 (165) | 60 (130) | 60 (140) |
| Homework time (hours/day), n (%) |  |  |  |  |
| 0 hours | 405 (1.2) | 620 (1.8) | 72 (0.9) | 162 (2) |
| <1 hour | 7,752 (22.2) | 10,537 (30.8) | 1,574 (19.5) | 2,511 (30.3) |
| 1-2 hours | 13,431 (38.4) | 12,978 (38) | 3,166 (39.3) | 3,203 (38.7) |
| 2-3 hours | 7,253 (20.7) | 5,510 (16.1) | 1,839 (22.8) | 1,380 (16.7) |
| ≥3 hours | 6,132 (17.5) | 4,520 (13.2) | 1,401 (17.4) | 1,023 (12.4) |
| Out-of-campus learning time (hours/day), n (%) |  |  |  |  |
| 0 hours | 23,325 (64.4) | 26,614 (75.6) | 4,432 (53.7) | 5,780 (68.6) |
| <1 hour | 3,379 (9.3) | 2,588 (7.3) | 699 (8.5) | 674 (8) |
| 1-2 hours | 3,761 (10.4) | 3,426 (9.7) | 1,182 (14.3) | 1,078 (12.8) |
| 2-3 hours | 2,535 (7) | 1,369 (3.9) | 862 (10.4) | 446 (5.3) |
| ≥3 hours | 3,213 (8.9) | 1,225 (3.5) | 1,079 (13.1) | 452 (5.4) |
| Secondary outcomes |  |  |  |  |
| Screen-viewing time (minutes/day), median (IQR) | 160 (230) | 180 (220) | 160 (220) | 160 (210) |
| Internet use time (minutes/day), median (IQR) | 90 (160) | 90 (170) | 90 (150) | 90 (150) |
| Meeting screen-viewing time recommendation, n (%) | 13,588 (36.1) | 12,755 (34.8) | 2,994 (35.1) | 3,064 (35.1) |
| Meeting regulatory requirement on homework time, n (%) | 16,060 (45.9) | 19,725 (57.7) | 3,058 (38) | 4,427 (53.5) |

Abbreviations: IQR, interquartile range; SD, standard deviation; GDP, gross domestic product

## Supplementary Table 19. Percentage changes in sedentary behaviours of participants taking part in both waves after the introduction of nationwide regulations (exploratory repeated cross-sectional analyses).

|  | Comparison between Wave 1 and Wave 2 | | | | Interaction effect (interaction with Wave) | | |
| --- | --- | --- | --- | --- | --- | --- | --- |
|  | n | Estimate | 95% CI | *p* value^a^ | Estimate | 95% CI | *p* value^a^ |
| Primary outcomes |  |  |  |  |  |  |  |
| Total sedentary behaviour time^b^ |  |  |  |  |  |  |  |
| Model 1^c^ | 76,831 | -10.4 | (-14.1, -6.7) | <0.001 |  |  |  |
| Model 2^d^ | 76,829 | -11.4 | (-15.1, -7.8) | <0.001 |  |  |  |
| Sex interaction^e^ | 76,829 | -11.6 | (-16.0, -7.2) | <0.001 | 0.4 | (-3.0, 3.7) | 0.83 |
| Education level interaction^f^ | 76,829 | -14.4 | (-20.2, -8.6) | <0.001 | 5.1 | (-2.4, 12.6) | 0.18 |
| Residency interaction^g^ | 76,829 | -8.1 | (-13.4, -2.7) | 0.0033 | -5.4 | (-12.7, 2.0) | 0.15 |
| Weight status interaction^h^ | 76,735 | -11.1 | (-14.8, -7.4) | <0.001 | -1.7 | (-4.8, 1.4) | 0.29 |
| Electronic device use time^i^ |  |  |  |  |  |  |  |
| Model 1^c^ | 84,997 | 5.7 | (-1.2, 12.5) | 0.11 |  |  |  |
| Model 2^d^ | 84,995 | 1.6 | (-5.3, 8.5) | 0.65 |  |  |  |
| Sex interaction^e^ | 84,995 | 2.6 | (-6.1, 11.4) | 0.56 | -2.02 | (-9.7, 5.7) | 0.61 |
| Education stage interaction^f^ | 84,995 | 4.8 | (-2.7, 12.2) | 0.21 | -5.36 | (-18.1, 7.4) | 0.41 |
| Residency interaction^g^ | 84,995 | 7.0 | (-5.2, 19.3) | 0.26 | -8.88 | (-24.0, 6.2) | 0.25 |
| Weight status interaction^h^ | 84,884 | 1.9 | (-5.2, 9.0) | 0.59 | -1.64 | (-8.2, 4.9) | 0.62 |
| Secondary outcomes |  |  |  |  |  |  |  |
| Total screen-viewing time^j^ |  |  |  |  |  |  |  |
| Model 1^c^ | 84,949 | -1.4 | (-6.1, 3.4) | 0.57 |  |  |  |
| Model 2^d^ | 84,947 | -2.0 | (-6.7, 2.6) | 0.39 |  |  |  |
| Sex interaction^e^ | 84,947 | -2.8 | (-8.9, 3.4) | 0.38 | 1.5 | (-4.0, 6.9) | 0.60 |
| Education stage interaction^f^ | 84,947 | 3.7 | (-1.1, 8.5) | 0.13 | -9.8 | (-18.2, -1.4) | 0.022 |
| Residency interaction^g^ | 84,947 | 1.2 | (-7.6, 9.9) | 0.80 | -5.2 | (-15.7, 5.3) | 0.33 |
| Weight status interaction^h^ | 84,836 | -2.3 | (-7.2, 2.5) | 0.35 | 1.5 | (-3.5, 6.5) | 0.56 |
| Internet use time^k^ |  |  |  |  |  |  |  |
| Model 1^c^ | 56,542 | 2.4 | (-6.4, 11.2) | 0.59 |  |  |  |
| Model 2^d^ | 56,540 | 1.1 | (-8.0, 10.1) | 0.82 |  |  |  |
| Sex interaction^e^ | 56,540 | 5.3 | (-5.8, 16.5) | 0.35 | -8.9 | (-20.0, 2.2) | 0.12 |
| Education level interaction^f^ | NA | NA | NA | NA | NA | NA | NA |
| Residency interaction^g^ | 56,540 | 9.3 | (-10.9, 29.5) | 0.37 | -12. | (-34.5, 10.6) | 0.30 |
| Weight status interaction^h^ | 56,494 | 0.8 | (-8.3, 9.9) | 0.86 | 1.5 | (-7.3, 10.4) | 0.73 |

Abbreviations: CI, confidence interval; NA, non-applicable.

^a^ Significance assessed at *p* < 0.005 using the Bonferroni correction.

^b^ Calculated in minutes as the sum of self-reported electronic device use time per day, TV/video game use time per day, computer use time per day, homework time, and out-of-campus learning time.

^c^ Unadjusted model.

^d^ Model 1 + age, sex, socioeconomic status, and area of residence.

^e^ Model 2 + Wave-sex (boys vs girls) interaction. Reference group are girls/Wave 1.

^f^ Model 2 + Wave-education stage (primary school vs secondary school) interaction. Reference group are primary school students/Wave 1.

^g^ Model 2 + Wave-residency (urban vs rural) interaction. Reference group are students living in rural areas/Wave 1.

^h^ Model 2 + Wave-weight status (normal weight vs overweight/obesity) interaction. Reference group are participants with normal weight/Wave 1.

^i^ Calculated in minutes from self-reported average time (hours and minutes) per day spent using mobile phones, handheld game consoles, and tablets.

^j^ Total screen-viewing time calculated as the sum of self-reported electronic device use time per day, TV/video game use time per day, and computer use time per day.

^k^ Self-reported average time (hours and minutes) per day spent ‘online’; only measured in secondary school students.

## Supplementary Table 20. Percentage changes in sedentary behaviours of participants after the introduction of nationwide regulations, boys in comparison to girls (exploratory repeated cross-sectional analyses).

|  | Boys | | | | Girls | | | |
| --- | --- | --- | --- | --- | --- | --- | --- | --- |
|  | n | Estimate | 95% CI | *p* value^a^ | n | Estimate | 95% CI | *p* value^a^ |
| Primary outcomes |  |  |  |  |  |  |  |  |
| Total sedentary behaviour time^b^ |  |  |  |  |  |  |  |  |
| Model 1^c^ | 39,017 | -10.5 | (-14.1, -6.9) | <0.001 | 38,041 | -11.0 | (-15.6, -6.4) | <0.001 |
| Model 2^d^ | 39,016 | -11.6 | (-15.2, -8.1) | <0.001 | 38,040 | -12.1 | (-16.5, -7.7) | <0.001 |
| Electronic device use time ^e^ |  |  |  |  |  |  |  |  |
| Model 1^c^ | 43,073 | 4.4 | (-2.3, 11.2) | 0.20 | 42,217 | 6.3 | (-2.7, 15.4) | 0.17 |
| Model 2^d^ | 43,072 | -0.9 | (-7.6, 5.8) | 0.80 | 42,216 | 2.4 | (-6.4, 11.3) | 0.59 |
| Secondary outcomes |  |  |  |  |  |  |  |  |
| Screen-viewing time^f^ | |  |  |  |  |  |  |  |
| Model 1^c^ | 43,047 | -0.9 | (-5.4, 3.6) | 0.70 | 42,194 | -2.6 | (-9.2, 4.1) | 0.45 |
| Model 2^d^ | 43,046 | -2.3 | (-6.6, 2.0) | 0.29 | 42,193 | -3.0 | (-9.4, 3.4) | 0.36 |
| Internet use time^g^ | |  |  |  |  |  |  |  |
| Model 1^c^ | 27,798 | -2.9 | (-12.6, 6.8) | 0.56 | 28,894 | 6.0 | (-5.3, 17.3) | 0.30 |
| Model 2^d^ | 27,797 | -4.9 | (-14.7, 4.9) | 0.32 | 28,893 | 5.1 | (-6.3, 16.5) | 0.38 |

Abbreviations: CI, confidence interval.

^a^ Significance assessed at *p* < 0.005 using the Bonferroni correction.

^b^ Calculated in minutes as the sum of self-reported electronic device use time per day, TV/video game use time per day, computer use time per day, homework time, and out-of-campus learning time.

^c^Unadjusted model.

^d^Model 1 + age, sex, socioeconomic status, and area of residence.

^e^Calculated in minutes from self-reported average time (hours and minutes) per day spent using mobile phones, handheld game consoles, and tablets.

^f^Calculated in minutes as the sum of self-reported electronic device use time per day, TV/video game use time per day, and computer use time per day.

^g^Calculated in minutes from self-reported average time (hours and minutes) per day spent ‘online’; only measured in secondary school children.

## Supplementary Table 21. Percentage changes in sedentary behaviours of participants after the introduction of nationwide regulations, primary in comparison to secondary school students (exploratory repeated cross-sectional analyses).

|  | Primary school students | | | | Secondary school students | | | |
| --- | --- | --- | --- | --- | --- | --- | --- | --- |
|  | n | Estimate | 95% CI | *p* value^a^ | n | Estimate | 95% CI | *p* value^a^ |
| Primary outcomes |  |  |  |  |  |  |  |  |
| Total sedentary behaviour time^b^ | |  |  |  |  |  |  |  |
| Model 1^c^ | 26,466 | -15.1 | (-21.7, -8.6) | <0.001 | 50,365 | -8.3 | (-12.8, -3.7) | <0.001 |
| Model 2^d^ | 26,466 | -20.9 | (-27.2, -14.7) | <0.001 | 50,363 | -6.6 | (-10.9, -2.3) | 0.0027 |
| Electronic device use time^e^ |  |  |  |  |  |  |  |  |
| Model 1^c^ | 28,359 | 14.2 | (3.5, 24.9) | 0.0092 | 56,638 | 2.3 | (-6.5, 11.1) | 0.61 |
| Model 2^d^ | 28,359 | -3.6 | (-13.8, 6.5) | 0.48 | 56,636 | 3.4 | (-5.1, 12.0) | 0.43 |
| Secondary outcomes |  |  |  |  |  |  |  |  |
| Screen-viewing time^f^ | |  |  |  |  |  |  |  |
| Model 1^c^ | 28,331 | 5.4 | (0.1, 10.6) | 0.045 | 56,618 | -5.5 | (-11.9, 0.9) | 0.092 |
| Model 2^d^ | 28,331 | -4.1 | (-9.1, 0.9) | 0.10 | 56,616 | -1.8 | (-8.0, 4.3) | 0.56 |
| Internet use time^g^ | |  |  |  |  |  |  |  |
| Model 1^c^ | NA | NA | NA | NA | NA | NA | NA | NA |
| Model 2^d^ | NA | NA | NA | NA | NA | NA | NA | NA |

Abbreviations: CI, confidence interval.

^a^Significance assessed at *p* < 0.005 using the Bonferroni correction.

^b^Calculated in minutes as the sum of self-reported electronic device use time per day, TV/video game use time per day, computer use time per day, homework time, and out-of-campus learning time.

^c^Unadjusted model.

^d^Model 1 + age, sex, socioeconomic status, and area of residence.

^e^Calculated in minutes from self-reported average time (hours and minutes) per day spent using mobile phones, handheld game consoles, and tablets.

^f^Calculated in minutes as the sum of self-reported electronic device use time per day, TV/video game use time per day, and computer use time per day.

^g^Calculated in minutes from self-reported average time (hours and minutes) per day spent ‘online’; only measured in secondary school children.

## Supplementary Table 22. Percentage changes in sedentary behaviours of participants after the introduction of nationwide regulations, urban in comparison to rural residents (exploratory repeated cross-sectional analyses).

|  | Urban residents | | | | Rural residents | | | |
| --- | --- | --- | --- | --- | --- | --- | --- | --- |
|  | n | Estimate | 95% CI | *p* value^a^ | n | Estimate | 95% CI | *p* value^a^ |
| Primary outcomes |  |  |  |  |  |  |  |  |
| Total sedentary behaviour time^b^ |  |  |  |  |  |  |  |  |
| Model 1^c^ | 46,842 | -12.1 | (-16.7, -7.5) | <0.001 | 29,989 | -7.4 | (-13.1, -1.6) | 0.012 |
| Model 2^d^ | 46,842 | -11.9 | (-16.5, -7.3) | <0.001 | 29,987 | -10.3 | (-15.7, -4.8) | <0.001 |
| Electronic device use time^e^ |  |  |  |  |  |  |  |  |
| Model 1^c^ | 51,373 | 2.3 | (-5.3, 9.9) | 0.56 | 33,624 | 11.8 | (-1.5, 25.1) | 0.081 |
| Model 2^d^ | 51,373 | 1.1 | (-6.7, 8.9) | 0.79 | 33,622 | 3.1 | (-10.1, 16.2) | 0.65 |
| Secondary outcomes |  |  |  |  |  |  |  |  |
| Screen-viewing time^f^ | |  |  |  |  |  |  |  |
| Model 1^c^ | 51,363 | -3.2 | (-8.4, 1.9) | 0.22 | 33,586 | 1.8 | (-7.5, 11.1) | 0.70 |
| Model 2^d^ | 51,363 | -2.3 | (-7.6, 2.9) | 0.39 | 33,584 | -1.4 | (-10.2, 7.4) | 0.76 |
| Internet use time^g^ | |  |  |  |  |  |  |  |
| Model 1^c^ | 36,463 | -1.1 | (-9.9, 7.8) | 0.81 | 20,079 | 11.1 | (-10.0, 32.3) | 0.30 |
| Model 2^d^ | 36,463 | -0.9 | (-9.9, 8.2) | 0.85 | 20,077 | 6.2 | (-15.7, 28.2) | 0.58 |

Abbreviations: CI, confidence interval.

^a^Significance assessed at *p* < 0.005 using the Bonferroni correction.

^b^Calculated in minutes as the sum of self-reported electronic device use time per day, TV/video game use time per day, computer use time per day, homework time, and out-of-campus learning time.

^c^Unadjusted model.

^d^Model 1 + age, sex, socioeconomic status, and area of residence.

^e^Calculated in minutes from self-reported average time (hours and minutes) per day spent using mobile phones, handheld game consoles, and tablets.

^f^Calculated in minutes as the sum of self-reported electronic device use time per day, TV/video game use time per day, and computer use time per day.

^g^Calculated in minutes from self-reported average time (hours and minutes) per day spent ‘online’; only measured in secondary school children.

## Supplementary Table 23. Percentage changes in sedentary behaviours of participants after the introduction of nationwide regulations, participant with normal weight in comparison to overweight/obesity (exploratory repeated cross-sectional analyses).

|  | Normal weight | | | | Overweight or obesity | | | |
| --- | --- | --- | --- | --- | --- | --- | --- | --- |
|  | n | Estimate | 95% CI | *p* value^a^ | n | Estimate | 95% CI | *p* value^a^ |
| Primary outcomes |  |  |  |  |  |  |  |  |
| Total sedentary behaviour time^b^ |  |  |  |  |  |  |  |  |
| Model 1^c^ | 62,080 | -9.6 | (-13.5, -5.7) | <0.001 | 14,657 | -13.6 | (-17.9, -9.4) | <0.001 |
| Model 2^d^ | 62,078 | -10.7 | (-14.4, -6.9) | <0.001 | 14,657 | -15.3 | (-19.7, -11.0) | <0.001 |
| Electronic device use time^e^ |  |  |  |  |  |  |  |  |
| Model 1^c^ | 68,929 | 6.4 | (-0.8, 13.7) | 0.083 | 15,957 | 2.5 | (-5.9, 10.9) | 0.56 |
| Model 2^d^ | 68,927 | 2.4 | (-4.8, 9.5) | 0.52 | 15,957 | -3.2 | (-11.7, 5.3) | 0.46 |
| Secondary outcomes |  |  |  |  |  |  |  |  |
| Screen-viewing time^f^ | |  |  |  |  |  |  |  |
| Model 1^c^ | 68,891 | -1.3 | (-6.4, 3.8) | 0.63 | 15,947 | -1.9 | (-8.0, 4.2) | 0.54 |
| Model 2^d^ | 68,889 | -1.9 | (-6.8, 3.0) | 0.44 | 15,947 | -3.6 | (-9.7, 2.4) | 0.24 |
| Internet use time^g^ | |  |  |  |  |  |  |  |
| Model 1^c^ | 47,347 | 2.5 | (-6.5, 11.5) | 0.58 | 9,139 | 2.1 | (-10.9, 15.0) | 0.75 |
| Model 2^d^ | 47,345 | 0.7 | (-8.5, 10.0) | 0.87 | 9,139 | 2.3 | (-10.1, 14.7) | 0.72 |

Abbreviations: CI, confidence interval.

^a^Significance assessed at *p* < 0.005 using the Bonferroni correction.

^b^Calculated in minutes as the sum of self-reported electronic device use time per day, TV/video game use time per day, computer use time per day, homework time, and out-of-campus learning time.

^c^Unadjusted model.

^d^Model 1 + age, sex, socioeconomic status, and area of residence.

^e^Calculated in minutes from self-reported average time (hours and minutes) per day spent using mobile phones, handheld game consoles, and tablets.

^f^Calculated in minutes as the sum of self-reported electronic device use time per day, TV/video game use time per day, and computer use time per day.

^g^Calculated in minutes from self-reported average time (hours and minutes) per day spent ‘online’; only measured in secondary school children.
